# An international RAND/UCLA expert panel to determine the optimal diagnosis and management of burn inhalation injury

**DOI:** 10.1186/s13054-023-04718-w

**Published:** 2023-11-27

**Authors:** Helena Milton-Jones, Sabri Soussi, Roger Davies, Emmanuel Charbonney, Walton N. Charles, Heather Cleland, Ken Dunn, Dashiell Gantner, Julian Giles, Marc Jeschke, Nicole Lee, Matthieu Legrand, Joanne Lloyd, Ignacio Martin-Loeches, Olivier Pantet, Mark Samaan, Odhran Shelley, Alice Sisson, Kaisa Spragg, Fiona Wood, Jeremy Yarrow, Marcela Paola Vizcaychipi, Andrew Williams, Jorge Leon-Villapalos, Declan Collins, Isabel Jones, Suveer Singh

**Affiliations:** 1 Faculty of Medicine, Imperial College London, London, UK; 2 Department of Anesthesia and Pain Management, Toronto Western Hospital, University Health Network, Toronto, ON Canada; 3 Inserm UMR-S 942, Cardiovascular Markers in Stress Conditions (MASCOT), University of Paris Cité, Paris, France; 4 Department of Intensive Care and Anaesthesia, Chelsea and Westminster Hospital NHS Foundation Trust, London, UK; 5 Department of Surgery and Cancer, Imperial College London, London, UK; 6 Intensive Care National Audit and Research Centre, London, UK; 7 Department of Médicine, Critical Care Division, Centre Hospitalier de l’Université de Montréal, Montréal, Canada; 8 Department of Medicine, Université de Montréal, Montréal, Canada; 9 Victorian Adult Burns Service, Alfred Health, Melbourne, Australia; 10 Department of Surgery, Central Clinical School, Monash University, Melbourne, Australia; 11 University Hospital South Manchester, Wythenshawe, UK; 12 Department of Intensive Care, Alfred Health, Melbourne, Australia; 13 Australian and New Zealand Intensive Care Research Centre, Monash University, Melbourne, Australia; 14 Department of Anaesthesia, Queen Victoria Hospital NHS Foundation Trust, East Grinstead, UK; 15 Ross Tilley Burn Center, Department of Surgery, Sunnybrook Health Science Center, Toronto, ON Canada; 16 Departments of Surgery and Immunology, University of Toronto, Toronto, ON Canada; 17 Department of Burns, Plastic and Reconstructive Surgery, Chelsea and Westminster Hospital NHS Foundation Trust, London, UK; 18 Department of Anesthesia and Perioperative Care, Division of Critical Care Medicine, University of California, San Francisco, USA; 19 Investigation Network Initiative-Cardiovascular and Renal Clinical Trialists Network, Nancy, France; 20 Department of Anaesthesia and Burns Intensive Care, St Andrew’s Centre for Burns and Plastic Surgery, Broomfield Hospital, Chelmsford, UK; 21 Department of Intensive Care Medicine, Multidisciplinary Intensive Care Research Organization (MICRO), St James Hospital, Dublin, Ireland; 22 Department of Respiratory Medicine, Hospital Clinic, IDIBAPS, CIBERes, Barcelona, Spain; 23 Universitat Barcelona, Barcelona, Spain; 24 Service of Adult Intensive Care, Lausanne University Hospital, Lausanne, Switzerland; 25 Department of Gastroenterology, University College London Hospitals NHS Foundation Trust, London, UK; 26 Trinity College, Dublin, Ireland; 27 Department of Plastic and Reconstructive Surgery, St James’ Hospital, Dublin, Ireland; 28 Burns Unit, Queen Victoria Hospital NHS Foundation Trust, East Grinstead, UK; 29 Fiona Stanley Hospital, Perth, Australia; 30 Perth Children’s Hospital, Perth, Australia; 31 University of Western Australia, Perth, Australia; 32 Welsh Centre for Burns and Plastic Surgery, Morriston Hospital, Swansea, UK; 33 Department of Research and Development, Chelsea and Westminster Hospital NHS Foundation Trust, London, UK; 34 Academic Department of Anaesthesia, Pain Management and Intensive Care (APMIC), Imperial College London, London, UK; 35 Royal Brompton Hospital, Guy’s and St Thomas’ Hospitals NHS Foundation Trust, London, UK

**Keywords:** Burn inhalation injury, Smoke inhalation injury, Burns, Acute lung injury, Acute respiratory distress syndrome, Bronchoscopy, Endotracheal intubation, Mechanical ventilation, Heparin

## Abstract

**Background:**

Burn inhalation injury (BII) is a major cause of burn-related mortality and morbidity. Despite published practice guidelines, no consensus exists for the best strategies regarding diagnosis and management of BII. A modified DELPHI study using the RAND/UCLA (University of California, Los Angeles) Appropriateness Method (RAM) systematically analysed the opinions of an expert panel. Expert opinion was combined with available evidence to determine what constitutes appropriate and inappropriate judgement in the diagnosis and management of BII.

**Methods:**

A 15-person multidisciplinary panel comprised anaesthetists, intensivists and plastic surgeons involved in the clinical management of major burn patients adopted a modified Delphi approach using the RAM method. They rated the appropriateness of statements describing diagnostic and management options for BII on a Likert scale. A modified final survey comprising 140 statements was completed, subdivided into history and physical examination (20), investigations (39), airway management (5), systemic toxicity (23), invasive mechanical ventilation (29) and pharmacotherapy (24). Median appropriateness ratings and the disagreement index (DI) were calculated to classify statements as appropriate, uncertain, or inappropriate.

**Results:**

Of 140 statements, 74 were rated as appropriate, 40 as uncertain and 26 as inappropriate. Initial intubation with ≥ 8.0 mm endotracheal tubes, lung protective ventilatory strategies, initial bronchoscopic lavage, serial bronchoscopic lavage for severe BII, nebulised heparin and salbutamol administration for moderate-severe BII and N-acetylcysteine for moderate BII were rated appropriate. Non-protective ventilatory strategies, high-frequency oscillatory ventilation, high-frequency percussive ventilation, prophylactic systemic antibiotics and corticosteroids were rated inappropriate. Experts disagreed (DI ≥ 1) on six statements, classified uncertain: the use of flexible fiberoptic bronchoscopy to guide fluid requirements (DI = 1.52), intubation with endotracheal tubes of internal diameter < 8.0 mm (DI = 1.19), use of airway pressure release ventilation modality (DI = 1.19) and nebulised 5000IU heparin, N-acetylcysteine and salbutamol for mild BII (DI = 1.52, 1.70, 1.36, respectively).

**Conclusions:**

Burns experts mostly agreed on appropriate and inappropriate diagnostic and management criteria of BII as in published guidance. Uncertainty exists as to the optimal diagnosis and management of differing grades of severity of BII. Future research should investigate the accuracy of bronchoscopic grading of BII, the value of bronchial lavage in differing severity groups and the effectiveness of nebulised therapies in different severities of BII.

**Graphical Abstract:**

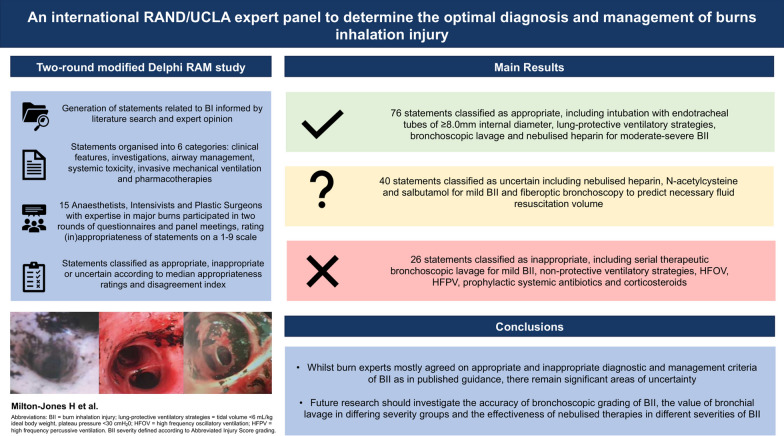

**Supplementary Information:**

The online version contains supplementary material available at 10.1186/s13054-023-04718-w.

## Background

Major burns are a global health problem and represent a significant proportion of patients treated in the intensive care unit (ICU). The World Health Organisation estimates 180,000 annual burn-related deaths worldwide, primarily occurring in household fires and workplace accidents [1]. One common burn-related manifestation is burn inhalation injury (BII), resulting from inhalation of hot gases and toxic substances in smoke [2]. The microscopic and systemic pathophysiological insult that manifests in the macroscopic tracheobronchitis of BII relates to the effects of a combination of processes. These include direct heat-related injury (pyrolysis and heated smoke-related particulate matter), oxygen deficit, local effects of toxins (e.g., reactive oxygen and nitrogen species and more soluble substances that predominantly affect the upper airway mucosa), and systemic effects of toxins (e.g., less soluble molecules that are absorbed and affect the tracheobronchial tree and/or parenchyma through epithelial/endothelial absorption) [2]. BII may occur in both the presence and absence of cutaneous burns [2]. BII has a prevalence of 19.8% among hospitalised burn patients and is an independent predictor of mortality, with an overall mortality rate of 10.9% [3–5]. In 1987, Shirani et al. reported that expected mortality in major burns patients rose by up to 20% in the presence of BII, and up to 60% when BII and pneumonia were both present [5]. Cutaneous burn patients with BII are at higher risk of developing earlier and more severe acute respiratory distress syndrome (ARDS) compared to those without BII [6]. BII survivors are faced with significant morbidity, including impaired exercise tolerance due to decreased respiratory muscle strength, cough capacity and forced vital capacity [7]. Socially, burn survivors with BII have significantly higher unemployment rates at 24 months post-injury than those without BII [8].

**Fig. 1:**
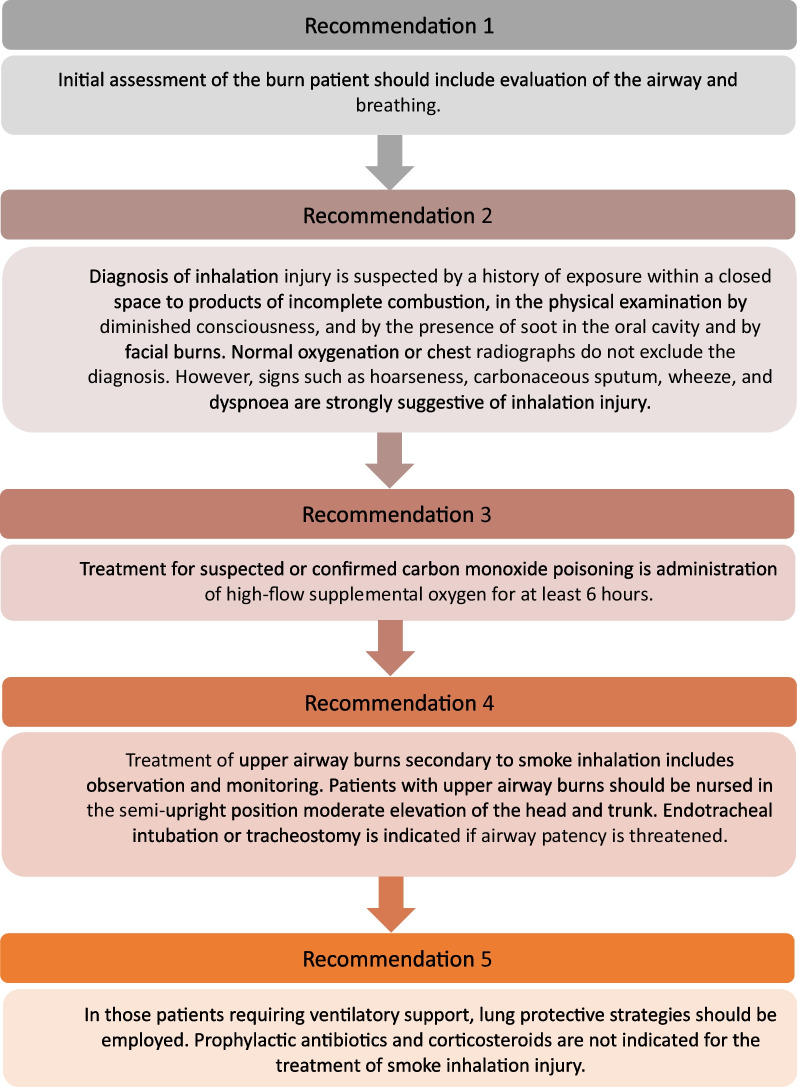
Current International Society for Burn Injuries (ISBI) practice guidelines for burn inhalation injury [9]

Despite the burden of BII, there remain uncertainties in the method of its diagnosis and management. Knowledge gaps exist due to a paucity of randomised control trials, and the 2016 International Society for Burn Injury (ISBI) practice guidelines for BII are based on high-quality but limited experimental data (Fig. 1) [9]. Features in the ISBI guidance include diagnosis being largely dependent on patient history and physical examination findings, whilst fibreoptic bronchoscopy (FOB) is considered to have great value in confirming initial diagnosis [10]. FOB, along with the Abbreviated Injury Score (AIS), can be used to grade BII severity [11]. Maintaining airway patency is emphasised in the guidelines, but the optimal tracheal tube size and timing of intubation or tracheostomy in BII remain unclear [9]. Many patients require invasive mechanical ventilation but the relationship between BII and ventilator-associated lung injury (VALI) is not well studied, and an evidence-based ventilatory strategy for BII is yet to be established [9]. Modern day practice assumes the use of low volume ventilation to protect against VALI as in ARDS, but its implementation remains variable and speculative. Targeted therapies under investigation include heparin to prevent fibrin clot formation, N-acetylcysteine to degrade mucus, a component of casts, and salbutamol as a bronchodilator [12]. Their relative efficacies and safeties are uncertain, as is the role, if any, of nebulised sodium bicarbonate, which does not have a published literature but has been reported to be practiced in some burns services in the UK [13].

Thus, we sought the opinions of a group of experts, using a pre-defined and structured methodology, combining available evidence and their own experience with the goal of investigating areas of most certainty in the management of patients affected by BII. A further aim was to identity areas of uncertainty or disagreement amongst experts, highlighting the need for improved practice guidance and further evidence.

## Methods

The RAND/University of California Los Angeles Appropriateness Method (RAM) uses a modified Delphi panel approach to combine expert opinion with available evidence to assess appropriateness of practices in defined clinical situations [14]. RAM is an internationally validated means of determining the benefit versus harm of a given intervention irrespective of cost or resources, particularly where robust evidence is lacking [14]. It has been successfully used in critical care for the management of ARDS precipitated by severe acute respiratory syndrome-coronavirus 2 infection [15].

A literature search on BII guided the creation of clinical vignettes representing scenarios in which BII may present. The search strategies relating to burn inhalation injury were combined with free text keywords and Medical Subject Headings relating to major themes in diagnosis and management. The following restrictions were applied: abstract available, full-text available, English language and human studies. Exclusion criteria included: animal and ex vivo studies, patients aged < 18 years and airway burns unrelated to fire and smoke inhalation such as steam, chemical, electrical and radiation burns. The clinical vignettes and statements outlining clinician management options were developed into the first-round questionnaire using the XM Qualtrics TM platform (*Waterloo, SE1 7ND London, UK*). BII severity was defined using established AIS grading (Additional file 1: Fig. S1).

A 15-member international, multidisciplinary panel of clinicians with expertise in BII was recruited through invitation following a search of burns services through international burn associations in Europe, North America, Australia and Asia during March and April 2022 (Additional file 2: Table S1). Panellists met eligibility criteria as consultant-level clinicians in Anaesthesia, Intensive Care Medicine, Plastic Surgery within specialist burns services. They had a minimum of 3 years of experience in diagnosing and managing adult patients with BII and demonstrated an interest in, and publications in, burns academia. To ensure balanced geographical representation, no more than two panellists were included per burn service. The steering committee also included senior burns service clinicians skilled in methodology, burns database development, and guideline formulation, who assisted in the study but who were not part of the panel (Additional file 3: Table S2).

Panellists received RAM instructions, a literature bibliography, and current ISBI guidelines. They rated the appropriateness of each statement in various clinical scenarios. Appropriateness was defined as ‘the procedure is anticipated to be more beneficial than harmful to the patient’. A 9-point scale was used for all questions, ranging from 1 (extremely inappropriate) to 9 (extremely appropriate).

Two virtual panel discussion meetings were conducted in April 2022 via Microsoft Teams. Panellists reviewed the results, discussing their interpretation of each statement and addressing areas of disagreement. Two Intensive Care nurses participated as non-voting experts to input BII nursing expertise. Both meetings were chaired by the same two moderators trained in RAM who abstained from expressing their opinions or voting.

The second-round questionnaire was developed using feedback from the panel. The questionnaire was organised into six chapters and 140 statements as follows: History and Physical Examination (20), Investigations (39), Airway Management (5), Systemic Toxicity (23), Invasive Mechanical Ventilation (29) and Pharmacotherapy (24).

### Statistical analysis

Statistical analysis was performed using Microsoft Excel (*Version 16.61, 2022*). Nonparametric outcome data were described as median. Each response on the 1–9 scale was recorded and a median appropriateness rating was calculated for each statement [14]. Median ratings of 7–9 were considered appropriate, 4–6 uncertain and 1–3 inappropriate [14, 15]. Subgroup analysis of the anaesthetists and intensivists (*n* = 10) was conducted for the Airway Management and Mechanical Ventilation chapters, as plastic surgeons reported limited expertise in these areas. To classify non-integer medians due to an even number of panellists, ratings of ≥ 6.5 were considered appropriate, < 6.5 and ≥ 3.5 as uncertain and < 3.5 as inappropriate [15].

The RAND disagreement index (DI) was calculated for each statement to assess panel disagreement (Eq. 1) [14]. The 30th–70th Interpercentile Range (IPR) was compared to the Interpercentile Range Adjusted for Symmetry (IPRAS) [14]. The IPRAS was calculated using the values best reproducing the classic definitions and are outlined in the RAND/UCLA Manual [14]. Disagreement was defined as DI ≥ 1 (where IPR ≥ IPRAS) and agreement as DI < 1 (where IPR < IPRAS) [14]. Statements that generated disagreement were classified as uncertain, regardless of the median panel rating [14].

1 $${\text{Disagreement}}\;{\text{index}}\;\left( {{\text{DI}}} \right) = \frac{{{\text{IPR}}}}{{{\text{IPRAS}}}} = \frac{{70{\text{th}} - 30{\text{th}}\;{\text{centile}}}}{{2.35 + \left( {1.5 \times {\text{abs}}\left( {5 - \frac{{70{\text{th}} + 30{\text{th}}\;{\text{centile}}}}{2}} \right)} \right)}}$$

Disagreement index (DI) for RAM statistical analysis, where abs is the absolute difference between the appropriateness score given and the panel median expressed as a positive number [14, 15].

### Ethical considerations

Patient-related ethical approval was not applicable to this study and so the Imperial College Research Ethics Committee waived any such requirement. All panellists provided informed consent prior to participation and consented to video and audio recordings of meetings.

## Results

The multidisciplinary panel comprised ten specialist in Anaesthesia and/or Intensive Care Medicine and five plastic surgeons with special interest in the management of major burn patients. Panellists were located across three continents in six countries: the UK, the Republic of Ireland, the USA, Canada, Switzerland and Australia. All 15 panellists completed both rounds. Demographic characteristics and clinical expertise of the panel are provided in Table 1.

**Table 1:** Demographic characteristics and clinical expertise of the RAM expert panel members (*n* = 15)

	Frequency	Percentage (%)
*Gender*		
Male	11	73.3
Female	4	26.7
*Age group (years)*		
65–74	1	6.7
55–64	2	13.3
45–54	6	40.0
35–44	6	40.0
*Country of residence*		
UK	5	33.3
Republic of Ireland	2	13.3
Switzerland	1	6.7
USA	1	6.7
Canada	3	20.0
Australia	3	20.0
*Specialty*		
Anaesthesia and/or Intensive Care Medicine	10	66.7
Plastic and Burns Surgery	5	33.3
*Current role in academia*		
Professor	5	33.3
Associate Professor	2	13.3
Senior Lecturer	2	13.3
Postdoctoral Researcher	1	6.7
Not applicable	5	33.3
*Years of burns service active clinical and/or academic work as of April 2022 (as a Consultant/Attending Physician)*		
30+	1	6.7
20–29	5	33.3
10–19	5	33.3
7–9	1	6.7
4–6	2	13.3
3	1	6.7

The final survey included 140 statements on BII diagnosis and management: 74 were rated appropriate, 40 uncertain, and 26 inappropriate. Agreement was reached for all scenarios except six. Disagreement arose regarding the use of FOB to guide fluid requirements, tracheal tube size for initial intubation, airway pressure release ventilation for ARDS, and the use of nebulised heparin, N-acetylcysteine, and salbutamol in mild BII.

### History and physical examination findings that lead to the suspicion of BII

**Fig. 2:**
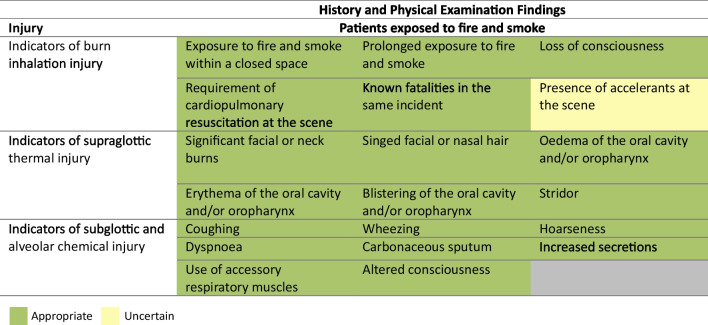
Appropriateness of using history and examination findings as indicators of potential burn inhalation injury. For each statement, median scores were calculated. Statements with a median score of ≤ 3 being classed inappropriate (red background), > 3 and < 7 uncertain (amber background) and ≥ 7 appropriate (green background). Disagreement was not present for any statements. Panellists *n* = 15

In patients with a history of exposure to fire and smoke, indicators including exposure to fire within a closed space, prolonged exposure, loss of consciousness, cardiopulmonary resuscitation and known fatalities in the same incident were rated as appropriate for suspecting a diagnosis of BII. The presence of accelerants at the scene was rated uncertain as an indicator. Physical examination findings of supraglottic injury including facial and neck burns, singed facial and nasal hair, oedema, erythema and blistering of the oral cavity and oropharynx, and stridor, were rated appropriate. Indicators of subglottic injury including coughing, wheezing, hoarseness, dyspnoea, carbonaceous sputum, increased secretions, the use of accessory respiratory muscles and altered consciousness were rated appropriate (Fig. 2).

### Investigations

To aid diagnosis of BII in acute and subacute settings, appropriate measures included arterial blood gas measurement (including lactate), carboxyhaemoglobin measurement, conventional and video laryngoscopy, fiberoptic nasendoscopy and FOB. Radionuclide imaging with ^133^xenon, magnetic resonance imaging (MRI) or pulmonary function tests were rated inappropriate. Point-of-care lung ultrasound, chest radiograph and chest computed tomography were rated uncertain. Of interest, chest radiograph was rated uncertain in the acute setting, which may be a non-significant statistical anomaly, however it represents a standard of practice for any hospital admission patient with major injuries.

**Fig. 3:**
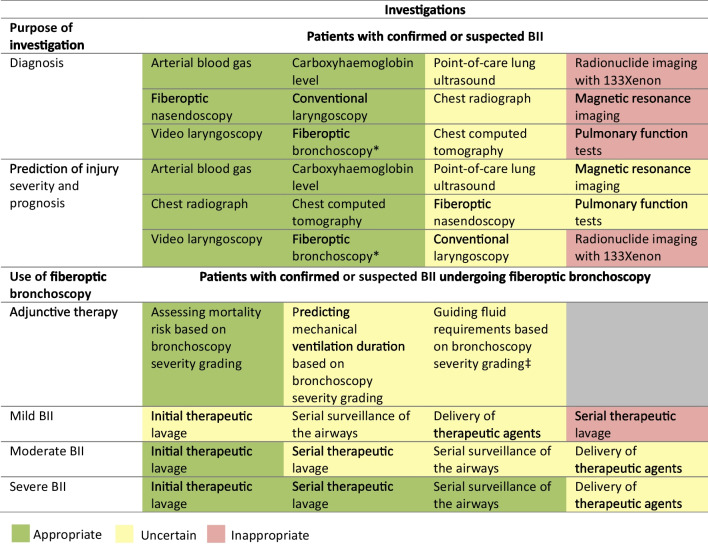
Appropriateness of investigations in the diagnosis and management of burn inhalation injury. For each statement, median scores were calculated. Statements with a median score of ≤ 3 being classed inappropriate (red background), > 3 and < 7 uncertain (amber background) and ≥ 7 appropriate (green background). Burn inhalation injury severity was defined according to Abbreviated Injury Score criteria as mild (grade 1), moderate (grade 2) and severe (grades 3–4). Disagreement was present for one statement (DI ≥ 1). BII, burn inhalation injury. Panellists *n* = 15. *Fiberoptic bronchoscopy, if intubated. ‡Denotes disagreement (DI ≥ 1)

To assess BII severity and prognosis in the acute or subacute setting, appropriate measures included arterial blood gas measurement, carboxyhaemoglobin measurement, chest radiograph, chest computed tomography, video laryngoscopy and FOB. Radionuclide imaging with ^133^xenon was rated inappropriate. This remains an experimental tool at present, hindered by the practicalities of clinical availability and access. Prognostication investigations after the acute diagnostic process, such as point-of-care lung ultrasound, fiberoptic nasendoscopy, conventional laryngoscopy, magnetic resonance imaging or pulmonary functions tests were rated uncertain (Fig. 3).

### FOB in the management of BII

FOB was rated appropriate in assessing mortality risk when used alongside AIS grading. Its use as an adjunct to guide fluid requirements generated disagreement and was rated uncertain (DI = 1.52). Uncertainty remained regarding its ability to predict duration of mechanical ventilation. FOB-initiated therapeutic lavage was rated uncertain for mild BII but appropriate in moderate and severe BII. Serial therapeutic lavage was rated inappropriate for mild BII, uncertain for moderate BII, and appropriate for severe BII. Serial visual assessment of the airways by FOB was uncertain for mild and moderate BII but appropriate for severe BII. The use of FOB to deliver therapeutic agents was rated uncertain across all BII severities (Fig. 3).

### Airway management

**Fig. 4:**
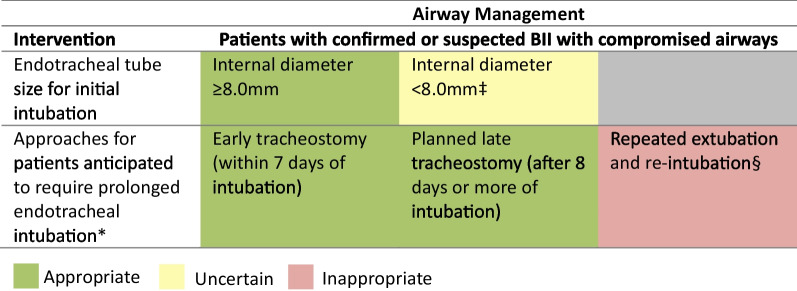
Appropriateness of airway management strategies for burn inhalation injury. For each statement, median scores were calculated. Statements with median score of < 3.5 were classed as inappropriate (red background), ≥ 3.5 and < 6.5 as uncertain (amber background) and ≥ 6.5 as appropriate (green background). Disagreement was present for one statement (DI ≥ 1). BII = burn inhalation injury. Panellists *n* = 10. *Exceeding 7 days. ‡Denotes disagreement (DI ≥ 1). §In-between theatre visits

Initial intubation with a standard cuffed endotracheal tube of internal diameter ≥ 8.0 mm was rated appropriate for BII patients at risk of airway compromise. Intubation with tracheal tubes < 8.0 mm generated disagreement and was rated uncertain (DI = 1.19). Repeated extubation and re-intubation between theatre trips for BII patients requiring surgery was rated inappropriate. Instead, early tracheostomy (within seven days of intubation) or late tracheostomy (after eight days or more of intubation) were rated appropriate (Fig. 4).

### Systemic toxicity in BII

**Fig. 5:**
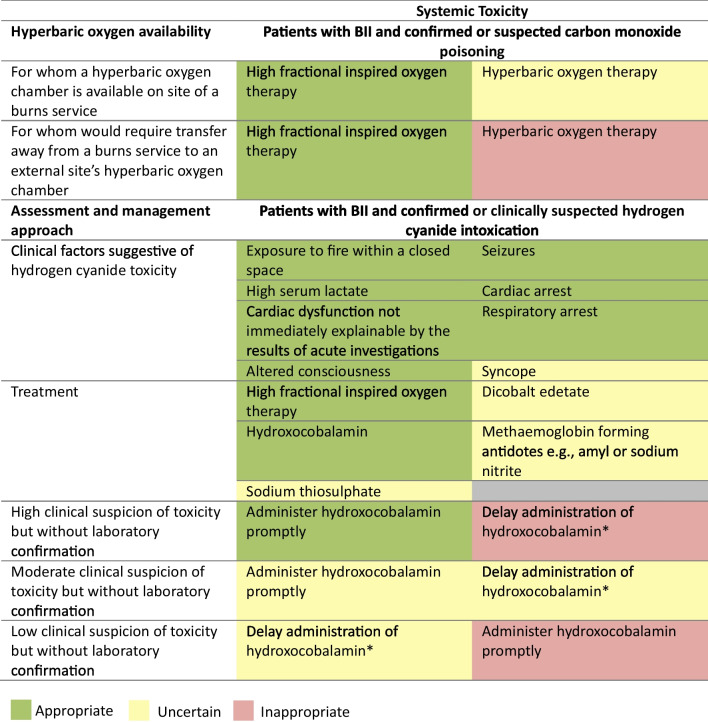
Appropriateness of diagnostic and management strategies for burn inhalation injury associated systemic toxicity. For each statement, median scores were calculated. Statements with a median score of ≤ 3 being classed inappropriate (red background), > 3 and < 7 uncertain (amber background) and ≥ 7 appropriate (green background). Clinical suspicion of hydrogen cyanide toxicity was defined as low (normal blood lactate and the absence of potentially suspicious features), moderate (moderate lactatemia below 8 mmol/L and few potentially suspicious features) and high (hyperlactataemia ≥ 8 mmol/L and potentially suspicious features including anion gap lactic metabolic acidosis, altered consciousness, unexplained cardiac dysfunction). High serum lactate was defined as ≥ 8 mmol/L. Disagreement was not present for any statements. BII, burn inhalation injury. Panellists *n* = 15. *Until a higher clinical suspicion or laboratory confirmation is available

For treatment of carbon monoxide intoxication, high fractional inspired oxygen therapy was rated appropriate. Patient transfer for hyperbaric oxygen therapy outside of a burns service was rated inappropriate. The theoretical availability of on-site hyperbaric oxygen therapy at a burns service was rated uncertain. Indicators such as exposure to fire and smoke within a closed space, raised serum lactate (≥ 8 mmol/L), unexplained cardiac dysfunction, altered consciousness, seizures, cardiac arrest and respiratory arrest were rated appropriate for suspecting hydrogen cyanide intoxication. Syncope as an indicator was rated uncertain. For treatment of hydrogen cyanide intoxication, high fractional inspired oxygen therapy and hydroxocobalamin were rated appropriate. Sodium thiosulphate, dicobalt edetate and amyl and sodium nitrite were rated uncertain. In the absence of laboratory confirmation of hydrogen cyanide intoxication, administering hydroxocobalamin promptly was rated appropriate for patients with hyperlactataemia ≥ 8 mmol/L, but inappropriate for those with normal lactate levels (< 2 mmol/L). It was rated inappropriate to delay administration of hydroxocobalamin to await laboratory confirmation of hydrogen cyanide toxicity for patients with hyperlactataemia. It remained uncertain whether hydroxocobalamin should be administered promptly for patients with only mild hyperlactataemia, or if administration should be delayed until laboratory confirmation or higher clinical suspicion of intoxication (Fig. 5).

### Invasive mechanical ventilation in BII

**Fig. 6:**
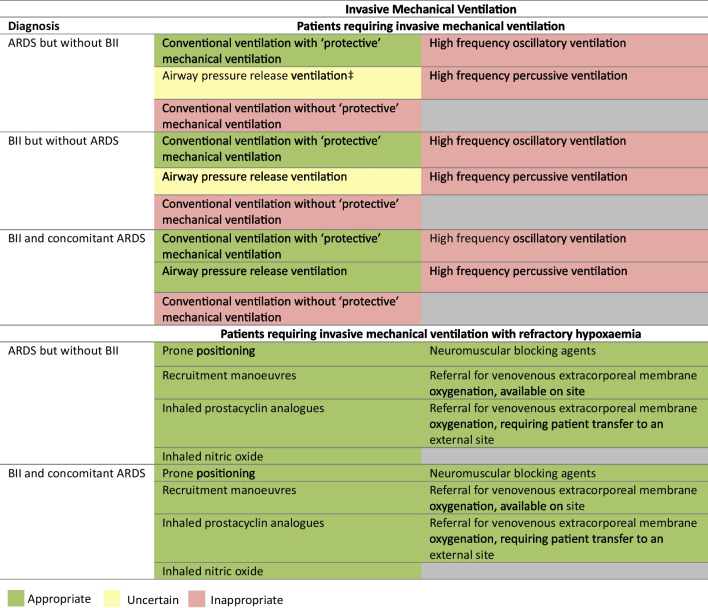
Appropriateness of ventilation strategies for burn inhalation injury and/or acute respiratory distress syndrome. For each statement, median scores were calculated. Statements with median score of < 3.5 were classed as inappropriate (red background), ≥ 3.5 and < 6.5 as uncertain (amber background) and ≥ 6.5 as appropriate (green background). Lung protective ventilatory strategies were defined as tidal volume < 6 mL/kg ideal body weight, plateau pressure < 30 cmH_2_0. Disagreement was present for one statement (DI ≥ 1). BII, burn inhalation injury. ARDS, acute respiratory distress syndrome. Panellists *n* = 10. ‡Denotes disagreement (DI ≥ 1)

For BII patients requiring mechanical ventilation both with and without ARDS, lung protective strategies (tidal volume < 6 mL/kg ideal body weight, plateau pressure < 30 cmH_2_0) were rated appropriate. Conventional non-lung protective ventilatory strategies, high-frequency oscillatory ventilation (HFOV) were rated inappropriate as was high-frequency percussive ventilation (HFPV), a ventilatory strategy used for effecting better airway clearance. Airway pressure release ventilation (APRV) was rated appropriate for BII patients with ARDS but uncertain for those without ARDS. Prone positioning, recruitment manoeuvres, inhaled prostacyclin analogues, inhaled nitric oxide, neuromuscular blocking agents and referral for venovenous extracorporeal membrane oxygenation (vvECMO) were rated appropriate for BII patients with refractory hypoxaemia. Referral for vvECMO was rated appropriate, even if it required transferring the patient from a burns service without vvECMO capabilities to an alternative ICU**.** The practicalities of separating the burns surgical and nursing teams from the ECMO ICU team are a challenge to optimal patient care, if not co-located (Fig. 6).

### Pharmacotherapy in BII

**Fig. 7:**
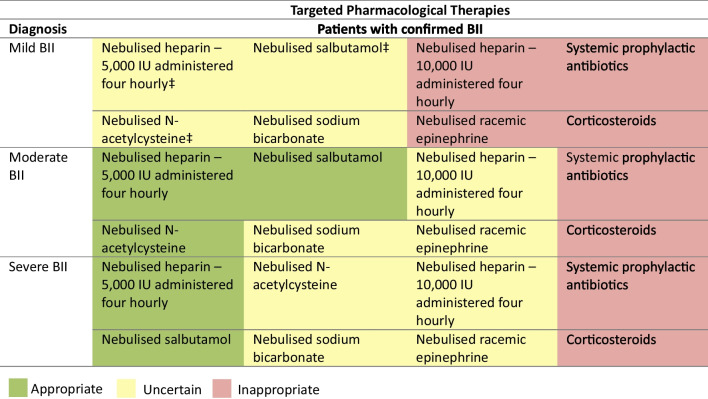
Appropriateness of pharmacological therapies for varying severities of burn inhalation injury. For each statement, median scores were calculated. Statements with a median score of ≤ 3 being classed inappropriate (red background), > 3 and < 7 uncertain (amber background) and ≥ 7 appropriate (green background). Burn inhalation injury severity was defined according to Abbreviated Injury Score criteria as mild (grade 1), moderate (grade 2) and severe (grades 3–4). Disagreement was present for three statements (DI ≥ 1). BII, burn inhalation injury. IU, international units. Panellists *n* = 15. ‡Denotes disagreement (DI ≥ 1)

5000 IU nebulised heparin was rated uncertain for patients with mild BII (DI = 1.52), but appropriate for moderate and severe BII. 10,000 IU nebulised heparin was rated inappropriate for mild BII and uncertain for moderate and severe BII. Nebulised N-acetylcysteine was rated uncertain for mild BII (DI = 1.70), appropriate for moderate BII and uncertain for severe BII. Nebulised sodium bicarbonate was rated uncertain regardless of BII severity. Nebulised salbutamol was rated uncertain for mild BII (DI = 1.36) and appropriate for moderate and severe BII. Nebulised racemic epinephrine was rated inappropriate for mild BII and uncertain for moderate and severe BII. Systemic prophylactic antibiotics and corticosteroids were rated inappropriate regardless of BII severity (Fig. 7).

**Fig. 8:**
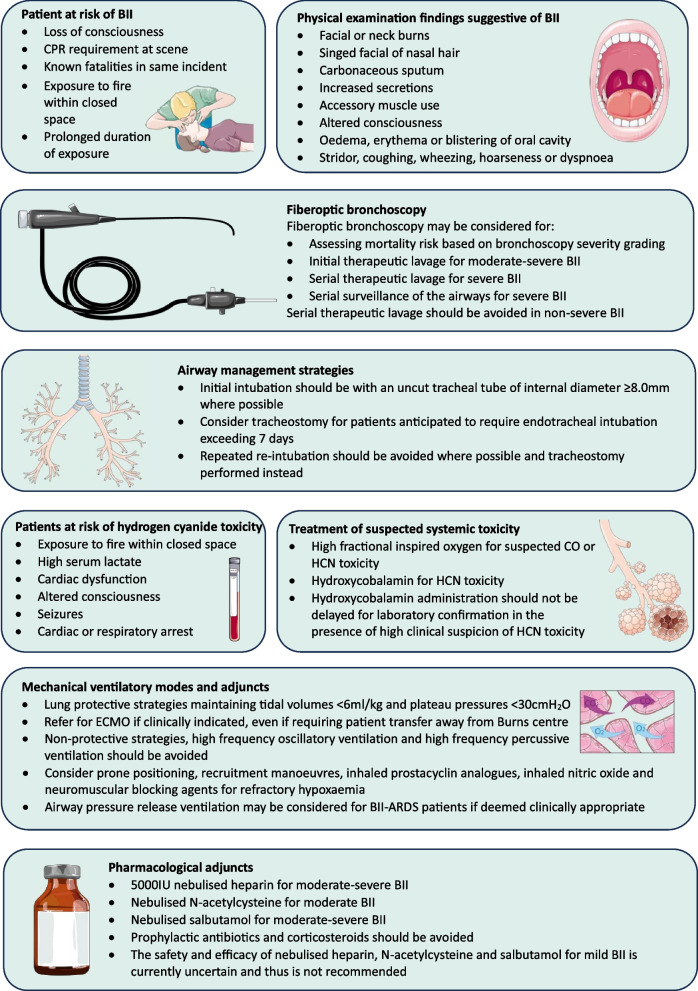
Summary of expert panel recommendations for burn inhalation injury. Parts of the figure were drawn by using pictures from Servier Medical Art. Servier Medical Art by Servier is licensed under a Creative Commons Attribution 3.0 Unported License (https://creativecommons.org/licenses/by/3.0/)

A complete table of the second-round questionnaire, median appropriateness ratings and disagreement index values for each statement are available in Additional file 4: Table S3. Detailed figures illustrating median scores and variation of the statement judgments are available as Additional file 5: Fig. S2, Additional file 6: Fig. S3, Additional file 7: Fig. S4, Additional file 8: Fig. S5, Additional file 9: Fig. S6, Additional file 10: Fig. S7. Key results and panel recommendations are summarised in Fig. 8.

## Discussion

Considering the paucity of contemporary evidence, this RAM study provides insight into the diagnosis and management of BII based on the opinions of an international panel of anaesthetists, intensivists and plastic surgeons from burns services around the world. Questions were constructed based on published guidelines with an aim to comprehensively capture clinically relevant issues in daily practice [9]. Whilst most areas of management were rated appropriate, and some clearly inappropriate, there remain significant areas of uncertainty amongst experts. These include aspects of FOB use, airway management, mechanical ventilation and nebulised pharmacological adjuncts for varying degrees of BII severity, such as heparin in mild BII.

The expert panel rated FOB as the most appropriate tool for confirming the initial diagnosis and assessing prognosis in BII. Previous studies have shown FOB to surpass reliance on history and clinical findings alone in diagnosing, evaluating severity and predicting outcomes in BII [16–18]. Thus, severe BII has been associated with increased mortality, while moderate BII is linked to prolonged mechanical ventilation [18]. A contrasting study reported that patients who underwent FOB on admission had higher mortality, higher pneumonia incidence, prolonged ICU stay, and duration of mechanical ventilation, raising concerns about potential iatrogenic injury [19]. This discrepancy may be due to clinicians performing FOB more frequently in severe BII cases, which inherently carry a greater risk of adverse outcomes unrelated to bronchoscopy itself. Nonetheless, it provides the degree of equipoise required for RAM methodology.

Regarding fluid strategy, there was uncertainty and significant disagreement as per FOB severity grading being predictive. Historical retrospective data indicate that burn injuries with BII require a higher fluid proportion than those without BII (5.0 ± 1.3 vs. 3.9 ± 0.9 mL/kg per % total body surface area [TBSA]), based on 3196 patients between 1980 and 2015 [20]. However, a study by Endorf and Gamelli found admission oxygenation status (partial pressure of arterial oxygen: fractional inspired oxygen concentration [P/F ratio]) was a better predictor of necessary fluid resuscitation volume than the AIS bronchoscopic grade [11]. Concerns regarding the impact of ‘fluid creep’ and variation in recorded delivered volumes have led to comparisons of crystalloid versus colloid fluid regimens on patient outcomes [20]. Current guidelines recommend 2–4 ml/kg/% TBSA as an estimated initial fluid volume with ongoing requirements guided by haemodynamic response and end organ perfusion as opposed to a BII-specific regime [9, 21].

The airway injury and subsequent inflammatory pathophysiology in BII can essentially be considered as various permutations of the upper airway/supraglottic mucosal injury, the tracheobronchitis, bronchiolitis and alveolitis [22]. Only the upper airway and tracheobronchial tree allow visual scrutiny. Thus, a gap exists in the tools to accurately demonstrate a causal link between the proximal airway injury and parenchymal injury, in the absence of an infective aetiology. Serial FOB lavage was rated appropriate for severe BII. In patients with major burns, BII and pneumonia, therapeutic lavage was associated with an 18% reduction in mortality based on a national US Burns registry study with over 9000 patients [23]. The expert panel did not support the need for serial lavage in mild cases of BII, presumably due to concerns about potential risks superseding the unlikely benefit of bronchoscopy for surveillance.

This expert panel’s findings align with other clinicians’ recommendations for transferring patients to burn services whilst intubated with an endotracheal tube of inner diameter at least 8.0 mm [24, 25]. The rationale behind this is that endotracheal tubes smaller than 8.0 mm do not allow for the passage of a bronchoscope with a sufficiently sized inner channel to allow effective bronchial toilet. Smaller endotracheal tubes also increase airflow resistance, compounding the intrinsic airways resistance and reduced lung compliance associated with acute lung injury/ARDS during positive pressure ventilation [25, 26]. Clinicians have reported an increase in patients arriving at burns services intubated with 7.0 mm or 7.5 mm endotracheal tubes [25]. Consequently, patients may require re-intubation with larger tubes to facilitate FOB, potentially delaying primary surgery and the risk of airway complications as well as being associated with greater duration of mechanical ventilation and ICU length of stay [27]. These findings suggest a practical approach to adopting the largest tube size possible in the early stages in BII patients. However, further research is required for clarification. At the very least, this should serve to raise awareness among first responders regarding critically ill patients suspected of having BII.

The experts rated highly the use of lung protective strategies, as those employed for ARDS patients, for critically ill patients with BII. These strategies that involve maintaining tidal volumes < 6 ml/kg and plateau pressures < 30 cmH_2_O, are widely adopted in ICU following the results of the pivotal ARDSNet trial [28]. The findings have since been extrapolated and applied to BII, with ISBI guidance recommending lung protective ventilation [9]. However, it should be noted that the ARDSNet trial did not include patients with significant burn injuries or report incidences of BII, limiting the generalisability of its findings to BII patients [28]. The unknown impact of smoke inhalation injury on VALI contributes to the challenge in developing an optimal ventilatory strategy [9]. Nonetheless, there is accruing evidence supporting the benefits of positive pressure ventilation within the critical care setting and even in perioperative medicine, of the benefits of a lung protective but volume guaranteed approach [29].

Whether BII patients should be ventilated differently to other critically ill patients has elicited the investigation of alternative ventilatory modes. The expert panel rated HFPV to be inappropriate despite previous, albeit limited work, which has supported its use [29, 30]. HFPV is reported historically to reduce ventilator-associated pneumonia compared to conventional mechanical ventilation in BII [30]. Another study found HFPV resulted in similar outcomes to low tidal volume ventilation for burn patients with respiratory failure [31]. The finding that the panel rated HFOV to be inappropriate in BII is consistent with the literature [32]. HFOV was reported to be less effective at improving oxygenation in ARDS burn patients who had co-existing BII compared to those without BII [33]. The findings of the OSCAR and OSCILLATE studies, which reported no survival advantage and potential harm in ARDS, may have influenced the panel’s judgement of inappropriateness for HFOV in BII [34, 35].

The panel’s rating of 5000 IU nebulised heparin as appropriate for moderate and severe BII aligns with evidence from preclinical and clinical studies [36–39]. The HIHI2 study and a recent meta-analysis of nine trials found that nebulised heparin (5000 IU or 10,000 IU) reduced hospital length of stay, and improved survival without major bleeding events [38, 39]. 10,000 IU has been reported to decrease lung injury scores and duration of mechanical ventilation compared to 5000 IU [40]. However, there is a lack of studies investigating the efficacy and safety of nebulised therapies for varying BII severities, which could explain the panel’s uncertain and inappropriate ratings.

As for hyperbaric oxygen therapy, there is no robust evidence base for its use in BII, and logistical challenges and risks associated with patient transfer likely contributed to the panel’s dismissal of any recommendation [41].

To our knowledge, this is the first consensus study to focus specifically on BII using this methodology. The calibre and inclusion of experts internationally is a strength of this study. There are several limitations that should be acknowledged. The panel lacked expertise from other countries and emergency medicine specialists who have an important role as first responders in the initial diagnosis and acute management of BII. Efforts were made to correct question ambiguity during panel discussions; however it is possible that some degree of misunderstanding of questions existed in the second-round questionnaire leading to uncertainty ratings. The panel’s interaction with literature and ISBI guidelines was not measured. Subgroup analysis of anaesthetists and intensivists rounded decimal median ratings of 3.5 and 6.5 up to form the boundaries of the uncertain and appropriate ratings. This method favoured classifying statements as appropriate [14]. An alternative approach could be to classify median ratings of > 6.5 appropriate, ≤ 6.5 and ≥ 3.5 uncertain and < 3.5 inappropriate to balance statement categorisation [14].

### Future research

Future research could explore associations between key inflammatory marker profiles, new methodologies such as volatilomics, and early-stage lung imaging to predict the development of ARDS and worse outcomes in BII. Developing bronchoscopic image registries could facilitate multimodal characterisation of BII and is desirable given the opportunities that may present themselves through predictive modelling using machine learning and artificial intelligence. Thus, allowing more accurate bronchoscopic severity grading and evaluation of the strength of its association with ARDS and mortality. There is also a need for future research to explore the efficacy and safety of nebulised therapies across different BII severities.

## Conclusion

This expert panel-modified Delphi RAM study is, to our knowledge, the first of its kind to focus specifically on BII and provides important insights and guidance for the diagnosis and management of BII. Future research should explore associations between key inflammatory marker profiles, new methodologies such as volatilomics, improved accuracy of bronchoscopic severity grading and early-stage lung imaging to predict the development of ARDS and worse outcomes in BII. The utilisation of artificial intelligence capabilities and further international collaboration on BII will enable such goals.

### Supplementary Information

**Additional file 1: Fig. S1**: Abbreviated Injury Score (AIS), adapted from Endorf et al. [20].

**Additional file 2: Table S1.** The RAND/UCLA Expert Panel.

**Additional file 3: Table S2.** The RAND/UCLA Steering Committee.

**Additional file 4: Table S3.** The second-round questionnaire complete with median appropriateness ratings and disagreement index values for each statement.

**Additional file 5: Fig. S2**. Appropriateness of using history and examination findings as indicators of potential burn inhalation injury. **A** Indicators of potential burn inhalation injury. **B** Indicators of potential supraglottic thermal injury. **C** Indicators of potential subglottic and alveolar chemical injury. Median ratings are presented (bold black line in each box) with the interquartile range (edges of box) and maximum and minimum ratings (extending vertical lines). Statements with median ratings of 1–3 were classed as inappropriate, 4–6 as uncertain and 7–9 as appropriate. Disagreement (disagreement index ≥ 1) was not present for any statements. Panellists *n* = 15. DI, disagreement index.

**Additional file 6: Fig. S3.** Appropriateness of investigations in the management of burn inhalation injury. **A** Investigations as diagnostic and prognostic tools for burn inhalation injury. **B** Using diagnostic fiberoptic bronchoscopy to predict outcomes in burn inhalation injury. **C** Using therapeutic fiberoptic bronchoscopy for varying severities of burn inhalation injury. Median ratings are presented (bold black line in each box) with the interquartile range (edges of box) and maximum and minimum ratings (extending vertical lines). Statements with median ratings of 1–3 were classed as inappropriate, 4–6 as uncertain and 7–9 as appropriate. *Disagreement (disagreement index ≥ 1) was present for one statement, which was classed as uncertain. Burn inhalation injury severity was defined according to Abbreviated Injury Score criteria as mild (grade 1), moderate (grade 2) and severe (grades 3–4). Panellists *n* = 15. BII, burn inhalation injury; DI, disagreement index.

**Additional file 7: Fig. S4**. Appropriateness of airway management strategies for burn inhalation injury. **A** Airway management strategies for burn inhalation injury patients at risk of airway compromise. **B** Airway management strategies for burn inhalation injury patients anticipated to require endotracheal intubation exceeding seven days. Median ratings are presented (bold black line in each box) with the interquartile range (edges of box) and maximum and minimum ratings (extending vertical lines). Statements with median ratings of < 3.5 were classed as inappropriate, ≥ 3.5 and < 6.5 as uncertain and ≥ 6.5 as appropriate. *Disagreement (disagreement index ≥ 1) was present for one statement, which was classed as uncertain. Panellists *n* = 10. DI, disagreement index.

**Additional file 8: Fig. S5**. Appropriateness of diagnostic and management strategies for burn inhalation injury associated systemic toxicity. **A** Management strategies for burn inhalation injury associated carbon monoxide toxicity. **B** Indicators of burn inhalation injury associated hydrogen cyanide toxicity. **C** Management strategies for burn inhalation injury associated hydrogen cyanide toxicity. **D** Timing of hydroxocobalamin administration for varying severities of burn inhalation injury. Median ratings are presented (bold black line in each box) with the interquartile range (edges of box) and maximum and minimum ratings (extending vertical lines). Statements with median ratings of 1–3 were classed as inappropriate, 4–6 as uncertain and 7–9 as appropriate. Disagreement (disagreement index ≥ 1) was not present for any statements. Clinical suspicion of hydrogen cyanide toxicity was defined as low (normal blood lactate and the absence of potentially suspicious features), moderate (moderate lactatemia below 8 mmol/L and few potentially suspicious features) and high (hyperlactataemia ≥ 8 mmol/L and potentially suspicious features including anion gap lactic metabolic acidosis, altered consciousness, unexplained cardiac dysfunction). Panellists *n* = 15. DI, disagreement index; HCN, hydrogen cyanide.

**Additional file 9: Fig. S6.** Appropriateness of ventilation strategies for burn inhalation injury and/or acute respiratory distress syndrome. **A** Invasive mechanical ventilation modalities for acute respiratory distress syndrome, burn inhalation injury or burn inhalation injury with concomitant acute respiratory distress syndrome. **B** Mechanical ventilation adjuncts for burn inhalation injury patients with refractory hypoxaemia. Median ratings are presented (bold black line in each box) with the interquartile range (edges of box) and maximum and minimum ratings (extending vertical lines). Statements with median ratings of < 3.5 were classed as inappropriate, ≥ 3.5 and < 6.5 as uncertain and ≥ 6.5 as appropriate. *Disagreement (disagreement index ≥ 1) was present for one statement, which was classed as uncertain. Lung protective ventilatory strategies = tidal volume < 6 mL/kg ideal body weight, plateau pressure < 30 cmH_2_0. Panellists *n* = 10. ARDS, acute respiratory distress syndrome; BII, burn inhalation injury; DI, disagreement index.

**Additional file 10: Fig. S7.** Appropriateness of pharmacological therapies for varying severities of burn inhalation injury. Median ratings are presented (bold black line in each box) with the interquartile range (edges of box) and maximum and minimum ratings (extending vertical lines). Statements with median ratings of 1–3 were classed as inappropriate, 4–6 as uncertain and 7–9 as appropriate. *Disagreement (disagreement index ≥ 1) was present for three statements, which were classed as uncertain. Burn inhalation injury severity was defined according to Abbreviated Injury Score criteria as mild (grade 1), moderate (grade 2) and severe (grades 3–4). Panellists *n* = 15. BII, burn inhalation injury; DI, disagreement index; IU, international units.

## Data Availability

The datasets analysed during the current study are available from the corresponding author on reasonable request.

## Abbreviations

ICU: Intensive care unit
BII: Burn inhalation injury
ARDS: Acute respiratory distress syndrome
ISBI: International society for burn injuries
FOB: Fiberoptic bronchoscopy
AIS: Abbreviated injury score
VALI: Ventilator-associated lung injury
RAM: RAND/University of California Los Angeles Appropriateness Method
DI: Disagreement index
IPR: Interpercentile range
IPRAS: Interpercentile range adjusted for symmetry
MRI: Magnetic resonance imaging
HFOV: High-frequency oscillatory ventilation
HFPV: High-frequency percussive ventilation
APRV: Airway pressure release ventilation
vvECMO: Venovenous extracorporeal membrane oxygenation
TBSA: Total body surface area
