# Supplementary material for: An international RAND/UCLA expert panel to determine the optimal diagnosis and management of burn inhalation injury
Source: Crit Care. 2023 Nov 27;27:459. doi: 10.1186/s13054-023-04718-w (PMC10680253; doi:10.1186/s13054-023-04718-w)
Supplement: Supplementary file 1 — Additional file 1: Fig. S1: Abbreviated Injury Score (AIS), adapted from Endorf et al. [20]. [file 13054_2023_4718_MOESM1_ESM.pdf]

Classification	Grade	Bronchoscopic criteria	Example
None	0	Absence of carbonaceous deposits, erythema, oedema, bronchorrhoea and obstruction	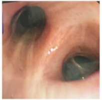
Mild	1	Minor/patchy areas of erythema and/or carbonaceous deposits in the proximal or distal bronchi	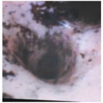
Moderate	2	Moderate degree of erythema, carbonaceous deposits and/or bronchorrhoea, with or without compromise of the bronchi	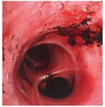
Severe	3	Severe inflammation with friability, copious carbonaceous deposits, bronchorrhoea and/or bronchial obstruction	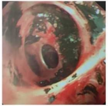
	4	Evidence of mucosal sloughing, necrosis and/or endoluminal obliteration	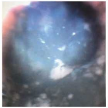
