# Supplementary material for: An international RAND/UCLA expert panel to determine the optimal diagnosis and management of burn inhalation injury
Source: Crit Care. 2023 Nov 27;27:459. doi: 10.1186/s13054-023-04718-w (PMC10680253; doi:10.1186/s13054-023-04718-w)
Supplement: Supplementary file 2 — Additional file 2: Table S1. The RAND/UCLA Expert Panel. [file 13054_2023_4718_MOESM2_ESM.docx]

| Panel Member | Role | Affiliations | Years of burns service active clinical and/or academic work as of April 2022 (as a Consultant/Burns Matron) |
| --- | --- | --- | --- |
| Professor Emmanuel Charbonney | Clinical Professor and Consultant in Intensive Care Medicine | Department of Medicine, Critical Care Division, Centre Hospitalier de l'Université de Montréal, Montréal, Canada; Department of Medicine, Université de Montréal, Montreal, QC, Canada. | 3 |
| Professor Heather Cleland | Clinical Professor and Consultant in Plastic Surgery;  Director of the Victorian Adult Burns Service, The Alfred Hospital | Victorian Adult Burns Service, Alfred Health, Melbourne, Australia; Department of Surgery, Central Clinical School, Monash University, Melbourne, Australia. | 20 |
| Dr Roger Davies | Consultant in Anaesthesia and Intensive Care | Department of Anaesthesia and Intensive Care, Chelsea and Westminster Hospital NHS Foundation Trust, London, United Kingdom. | 5 |
| Dr Dashiell Gantner | Consultant in Intensive Care Medicine | Department of Intensive Care, Alfred Health, Melbourne, Australia; Australian and New Zealand Intensive Care Research Centre, Monash University, Melbourne, Australia. | 11 |
| Dr Julian Giles | Consultant in Anaesthesia | Department of Anaesthesia, Queen Victoria Hospital NHS Foundation Trust, East Grinstead, UK. | 20 |
| Professor Marc Jeschke | Clinical Professor and Consultant Plastic and Burn Surgeon | Ross Tilley Burn Center, Department of Surgery, Sunnybrook Health Science Center, Toronto, Ontario, Canada; Departments of Surgery and Immunology, University of Toronto, Toronto, Ontario, Canada. | 26 |
| Professor Matthieu Legrand | Clinical Professor and Consultant in Anaesthesia and Intensive Care Medicine | Department of Anesthesia and Perioperative Care, Division of Critical Care Medicine, University of California, San Francisco, CA, USA; Investigation Network Initiative-Cardiovascular and Renal Clinical Trialists network, Nancy, France. | 10 |
| Dr Joanne Lloyd | Consultant in Anaesthesia and Intensive Care | Department of Anaesthesia, St Andrew's Centre for Plastic Surgery and Burns, Broomfield Hospital, Chelmsford, UK. | 22 |
| Professor Ignacio Martin-Loeches | Clinical Professor and Consultant in Intensive Care Medicine | Department of Intensive Care Medicine, Multidisciplinary Intensive Care Research Organization (MICRO), St James Hospital, Dublin 8, Ireland; Department of Respiratory Medicine, Hospital Clinic, IDIBAPS, CIBERes, Barcelona, Spain. | 15 |
| Dr Olivier Pantet | Consultant in Intensive Care Medicine | Service of Adult Intensive Care, Lausanne University Hospital (CHUV), Lausanne, Switzerland; Faculty of Biology & Medicine, Lausanne University, Lausanne, Switzerland. | 11 |
| Professor Odhran Shelley | Clinical Professor and Consultant in Plastic and Burn Surgery;  Director of the National Burn Unit, Dublin, Ireland | Department of Plastic and Reconstructive Surgery, St James' Hospital, Dublin 8, Ireland. | 27 |
| Dr Alice Sisson | Consultant in Anaesthesia and Intensive Care | Department of Anaesthesia and Intensive Care, Chelsea and Westminster Hospital NHS Foundation Trust, London, United Kingdom. | 6 |
| Professor Sabri Soussi | Consultant in Anaesthesia and Intensive Care Medicine | Department of Anesthesiology and Pain Medicine, University of Toronto, and the Department of Anesthesia and Pain Management, Toronto Western Hospital, University Health Network, Toronto, Ontario, Canada. | 8 |
| Professor Fiona Wood | Clinical Professor and Consultant in Plastic Surgery;  Director of the Burns Service of Western Australia | Fiona Stanley Hospital, Perth, Western Australia; Perth Children's Hospital, Perth, Western Australia; University of Western Australia, Australia. | 42 |
| Mr Jeremy Yarrow | Consultant in Plastic and Burn Surgery; Clinical Lead for Welsh Centre for Burns | Welsh Centre for Burns and Plastic Surgery, Morriston Hospital, Swansea, UK. | 10 |
