# Supplementary material for: An international RAND/UCLA expert panel to determine the optimal diagnosis and management of burn inhalation injury
Source: Crit Care. 2023 Nov 27;27:459. doi: 10.1186/s13054-023-04718-w (PMC10680253; doi:10.1186/s13054-023-04718-w)
Supplement: Supplementary file 3 — Additional file 3: Table S2. The RAND/UCLA Steering Committee. [file 13054_2023_4718_MOESM3_ESM.docx]

| Steering Committee Member | Role | Affiliations | Years of burns service active clinical and/or academic work as  of April 2022 (as a Consultant/Burns Matron) |
| --- | --- | --- | --- |
| Professor Suveer Singh | Clinical Professor and Consultant in Respiratory and Intensive Care Medicine | Chelsea & Westminster Hospital, Imperial College London | 20 |
| Dr Marcela Paola Vizcaychipi | Consultant in Anaesthesia and Intensive Care Medicine | Chelsea & Westminster Hospital, Imperial College London | 13 |
| Mr Andrew Williams | Consultant Burns and Plastic Surgeon | Chelsea & Westminster Hospital | 15 |
| Mr Jorge Leon-Villapalos | Consultant Burns and Plastic Surgeon | Chelsea & Westminster Hospital, Imperial College London | 13 |
| Mr Declan Collins | Consultant Burns and Plastic Surgeon | Chelsea & Westminster Hospital, Imperial College London | 7 |
| Ms Isabel Jones | Consultant Burns and Plastic Surgeon | Chelsea & Westminster Hospital, Imperial College London | 14 |
| Mr Ken Dunn | [Retired] Consultant Burns and Plastic Surgeon | University Hospital South Manchester, Wythenshawe, United Kingdom | 27 |
| Ms Nicole Lee | Burns Matron; Lead Nurse London and South East Burns Network | Chelsea and Westminster Hospital NHS Foundation Trust | 2 as Burns Matron; 17 years in burn care total |
| Dr Walton N Charles | National Institute for Health and Care Research (NIHR) Pre-doctoral Fellow in Epidemiology | Intensive Care National Audit and Research Centre, London; Imperial College London | N/A |
| Miss Helena Milton-Jones | Medical Student (MBBS/BSc) | Imperial College London | N/A |
