# Supplementary material for: An international RAND/UCLA expert panel to determine the optimal diagnosis and management of burn inhalation injury
Source: Crit Care. 2023 Nov 27;27:459. doi: 10.1186/s13054-023-04718-w (PMC10680253; doi:10.1186/s13054-023-04718-w)
Supplement: Supplementary file 4 — Additional file 4: Table S3. The second-round questionnaire complete with median appropriateness ratings and disagreement index values for each statement. [file 13054_2023_4718_MOESM4_ESM.docx]

| **Statement** | **Median rating** | **Disagreement index** | **Classification** |
| --- | --- | --- | --- |
| **Chapter 1: History and Physical Examination** | | | |
| **For patients exposed to fire, please rate the appropriateness of using the following factors in their history as indicators of potential burn inhalation injury:** | | | |
| Exposure to fire and smoke within a closed space | 9 | 0.10 | Appropriate |
| Prolonged exposure to fire and smoke | 9 | 0.26 | Appropriate |
| Loss of consciousness | 8 | 0.26 | Appropriate |
| Requirement of cardiopulmonary resuscitation at the scene | 8 | 0.13 | Appropriate |
| Known fatalities in the same incident | 8 | 0.16 | Appropriate |
| Presence of accelerants at the scene e.g., fuel or vehicle fire | 6 | 0.35 | Uncertain |
| **For patients exposed to fire, please rate the appropriateness of using the following factors in their physical examination as indicators of potential supraglottic thermal injury:** | | | |
| Significant facial or neck burns | 8 | 0.26 | Appropriate |
| Singed facial or nasal hair | 7 | 0.16 | Appropriate |
| Oedema of the oral cavity and/or oropharynx | 9 | 0.13 | Appropriate |
| Erythema of the oral cavity and/or oropharynx | 7 | 0.27 | Appropriate |
| Blistering of the oral cavity and/or oropharynx | 9 | 0.13 | Appropriate |
| Stridor | 9 | 0.13 | Appropriate |
| **For patients exposed to fire, please rate the appropriateness of using the following factors in their physical examination as indicators of potential subglottic and alveolar chemical injury:** | | | |
| Coughing | 7 | 0.16 | Appropriate |
| Wheezing | 8 | 0.16 | Appropriate |
| Hoarseness | 8 | 0.16 | Appropriate |
| Dyspnoea | 8 | 0.16 | Appropriate |
| Carbonaceous sputum | 8 | 0.13 | Appropriate |
| Increased secretions | 7 | 0.30 | Appropriate |
| Use of accessory respiratory muscles | 8 | 0.13 | Appropriate |
| Altered consciousness | 8 | 0.13 | Appropriate |
| **Chapter 2: Investigations** | | | |
| **In patients with suspected burn inhalation injury, please rate the appropriateness of the following investigations to aid the diagnosis of burn inhalation injury, either in the acute or subacute setting:** | | | |
| Arterial blood gas, including lactate measurement | 8 | 0.00 | Appropriate |
| Carboxyhaemoglobin level | 8 | 0.13 | Appropriate |
| Point-of-care lung ultrasound | 5 | 0.68 | Uncertain |
| Chest radiograph | 6 | 0.52 | Uncertain |
| Chest computed tomography | 5 | 0.49 | Uncertain |
| Fiberoptic nasendoscopy | 8 | 0.16 | Appropriate |
| Conventional laryngoscopy | 7 | 0.37 | Appropriate |
| Video laryngoscopy | 8 | 0.33 | Appropriate |
| Fiberoptic bronchoscopy, if intubated | 9 | 0.10 | Appropriate |
| Radionuclide imaging with ^133^Xenon | 3 | 0.37 | Inappropriate |
| Magnetic resonance imaging | 3 | 0.47 | Inappropriate |
| Pulmonary function tests | 3 | 0.22 | Inappropriate |
| **In patients with burn inhalation injury, please rate the appropriateness of the following investigations to predict injury severity and prognosis, either in the acute or subacute setting:** | | | |
| Arterial blood gas, including lactate measurement | 8 | 0.23 | Appropriate |
| Carboxyhaemoglobin measurement | 8 | 0.43 | Appropriate |
| Point-of-care lung ultrasound | 4 | 0.32 | Uncertain |
| Chest radiograph | 7 | 0.45 | Appropriate |
| Chest computed tomography | 7 | 0.94 | Appropriate |
| Fiberoptic nasendoscopy | 6 | 0.22 | Uncertain |
| Conventional laryngoscopy | 6 | 0.86 | Uncertain |
| Video laryngoscopy | 7 | 0.86 | Appropriate |
| Fiberoptic bronchoscopy, if intubated | 9 | 0.13 | Appropriate |
| Radionuclide imaging with ^133^Xenon | 3 | 0.37 | Inappropriate |
| Magnetic resonance imaging | 4 | 0.47 | Uncertain |
| Pulmonary function tests | 4 | 0.52 | Uncertain |
| **For all patients with burn inhalation injury, please rate the appropriateness of the following uses of fiberoptic bronchoscopy:** | | | |
| As an adjunct to guiding fluid requirements based on bronchoscopy severity grading | 6 | 1.52 | Uncertain |
| As an adjunct to predicting mechanical ventilation duration based on bronchoscopy severity grading | 6 | 0.45 | Uncertain |
| As an adjunct to assessing mortality risk based on bronchoscopy severity grading | 7 | 0.16 | Appropriate |
| **For patients with mild burn inhalation injury, (e.g., graded 1 using the Abbreviated Injury Score or an equivalent grade on a similar scoring system), please rate the appropriateness of the following uses of fiberoptic bronchoscopy:** | | | |
| Initial therapeutic lavage | 6 | 0.86 | Uncertain |
| Serial therapeutic lavage | 3 | 0.37 | Inappropriate |
| Serial surveillance of the airways | 5 | 0.32 | Uncertain |
| Delivery of therapeutic agents | 4 | 0.49 | Uncertain |
| **For patients with moderate burn inhalation injury, (e.g., graded 2 using the Abbreviated Injury Score or an equivalent grade on a similar scoring system), please rate the appropriateness of the following uses of fiberoptic bronchoscopy:** | | | |
| Initial therapeutic lavage | 7 | 0.62 | Appropriate |
| Serial therapeutic lavage | 6 | 0.95 | Uncertain |
| Serial surveillance of the airways | 6 | 0.45 | Uncertain |
| Delivery of therapeutic agents | 5 | 0.93 | Uncertain |
| **For patients with severe burn inhalation injury, (e.g., graded 3 or 4 using the Abbreviated Injury Score or an equivalent grade on a similar scoring system), please rate the appropriateness of the following uses of fiberoptic bronchoscopy:** | | | |
| Initial therapeutic lavage | 9 | 0.29 | Appropriate |
| Serial therapeutic lavage | 7 | 0.33 | Appropriate |
| Serial surveillance of the airways | 7 | 0.16 | Appropriate |
| Delivery of therapeutic agents | 6 | 0.63 | Uncertain |
| **Chapter 3: Airway Management** | | | |
| **For patients with clinically suspected burn inhalation injury presenting with an airway at risk of compromise, please rate the appropriateness of the following approaches:** | | | |
| Intubation with an uncut tracheal tube of internal diameter <8.0mm | 5 | 1.19 | Uncertain |
| Intubation with an uncut tracheal tube of internal diameter ≥8.0 mm | 8 | 0.00 | Appropriate |
| **For patients with confirmed burn inhalation injury who are anticipated to have prolonged endotracheal intubation (exceeding 7 days), please rate the appropriateness of the following approaches:** | | | |
| Repeated extubation and re-intubation | 3 | 0.68 | Inappropriate |
| Early tracheostomy (within 7 days of intubation) | 6.5 | 0.35 | Appropriate |
| Planned late tracheostomy (after 8 days or more of intubation) | 7 | 0.35 | Appropriate |
| **Chapter 4: Systemic Toxicity** | | | |
| **For burn inhalation injury patients with clinically suspected or confirmed carbon monoxide poisoning, for whom a hyperbaric oxygen chamber is available on site of a burns unit, please rate the appropriateness of administering the following treatments:** | | | |
| High fractional inspired oxygen therapy | 9 | 0.13 | Appropriate |
| Hyperbaric oxygen therapy | 4 | 0.52 | Uncertain |
| **For burn inhalation injury patients with clinically suspected or confirmed carbon monoxide poisoning, for whom would require transfer to an external site's hyperbaric oxygen chamber away from a burns unit, please rate the appropriateness of administering the following treatments:** | | | |
| High fractional inspired oxygen therapy | 9 | 0.13 | Appropriate |
| Hyperbaric oxygen therapy | 2 | 0.26 | Inappropriate |
| **For patients exposed to fire, please rate the appropriateness of using the following factors in their history and physical examination as indicators of potential cyanide intoxication:** | | | |
| Exposure within a closed space | 8 | 0.13 | Appropriate |
| High serum lactate (e.g., ≥ 8mmol/L) | 9 | 0.13 | Appropriate |
| Cardiac dysfunction not immediately explainable by the results of acute investigations e.g., echocardiogram | 8 | 0.27 | Appropriate |
| Altered consciousness | 8 | 0.16 | Appropriate |
| Syncope | 6 | 0.22 | Uncertain |
| Seizures | 7 | 0.22 | Appropriate |
| Cardiac arrest | 7 | 0.37 | Appropriate |
| Respiratory arrest | 8 | 0.33 | Appropriate |
| **For burn inhalation injury patients with confirmed or clinically suspected cyanide intoxication, please rate the appropriateness of administering the following treatments:** | | | |
| High fractional inspired oxygen therapy | 8 | 0.13 | Appropriate |
| Hydroxocobalamin | 9 | 0.10 | Appropriate |
| Sodium thiosulphate | 6 | 0.84 | Uncertain |
| Dicobalt edetate | 5 | 0.72 | Uncertain |
| Methaemoglobin forming antidotes e.g., amyl or sodium nitrite | 5 | 0.32 | Uncertain |
| **For burn inhalation injury patients with high clinical suspicion of cyanide toxicity (e.g., hyperlactataemia, altered consciousness, unexplained cardiac dysfunction) but without laboratory confirmation, please rate the appropriateness of the following approaches:** | | | |
| Administer hydroxocobalamin promptly | 9 | 0.10 | Appropriate |
| Delay administration of hydroxocobalamin until laboratory confirmation is available | 3 | 0.69 | Inappropriate |
| **For burn inhalation injury patients with moderate clinical suspicion of cyanide toxicity (e.g., moderate lactataemia and other potentially suspicious features) but without laboratory confirmation, please rate the appropriateness of the following approaches:** | | | |
| Administer hydroxocobalamin promptly | 6 | 0.65 | Uncertain |
| Delay administration of hydroxocobalamin until laboratory confirmation is available or you have a higher clinical suspicion | 6 | 0.97 | Uncertain |
| **For burn inhalation injury patients with low clinical suspicion of cyanide toxicity (e.g., normal lactataemia and the absence of potentially suspicious features) and without laboratory confirmation, please rate the appropriateness of the following approaches:** | | | |
| Administer hydroxocobalamin promptly | 3 | 0.43 | Inappropriate |
| Delay administration of hydroxocobalamin until laboratory confirmation is available or you have a higher clinical suspicion | 6 | 0.69 | Uncertain |
| **Chapter 5: Invasive Mechanical Ventilation** | | | |
| **For critically ill patients meeting ARDS diagnostic criteria but without burn inhalation injury requiring mechanical ventilation, please rate the appropriateness of the following mechanical ventilatory modes:** | | | |
| Conventional ventilation without ‘protective’ mechanical ventilation | 1.5 | 0.18 | Inappropriate |
| Conventional ventilation with ‘protective’ mechanical ventilation | 9 | 0.04 | Appropriate |
| High frequency oscillatory ventilation as rescue therapy | 2 | 0.56 | Inappropriate |
| High frequency percussive ventilation as rescue therapy | 1.5 | 0.41 | Inappropriate |
| Airway pressure release ventilation as rescue therapy | 6.5 | 1.19 | Uncertain |
| **For critically ill patients with burn inhalation injury requiring mechanical ventilation but not meeting ARDS diagnostic criteria, please rate the appropriateness of the following mechanical ventilatory strategies:** | | | |
| Conventional ventilation without ‘protective’ mechanical ventilation | 3 | 0.60 | Inappropriate |
| Conventional ventilation with ‘protective’ mechanical ventilation | 8.5 | 0.29 | Appropriate |
| High frequency oscillatory ventilation as rescue therapy | 1.5 | 0.41 | Inappropriate |
| High frequency percussive ventilation as rescue therapy | 2 | 0.41 | Inappropriate |
| Airway pressure release ventilation as rescue therapy | 4 | 0.89 | Uncertain |
| **For critically ill patients with burn inhalation injury and concomitant ARDS requiring mechanical ventilation, please rate the appropriateness of the following mechanical ventilatory strategies:** | | | |
| Conventional ventilation without ‘protective’ mechanical ventilation | 1 | 0.04 | Inappropriate |
| Conventional ventilation with ‘protective’ mechanical ventilation | 9 | 0.00 | Appropriate |
| High frequency oscillatory ventilation as rescue therapy | 2 | 0.41 | Inappropriate |
| High frequency percussive ventilation as rescue therapy | 2 | 0.75 | Inappropriate |
| Airway pressure release ventilation as rescue therapy | 6.5 | 0.78 | Appropriate |
| **For critically ill patients meeting ARDS diagnostic criteria but without burn inhalation injury receiving mechanical ventilation and experiencing refractory hypoxaemia, please rate the appropriateness of the following adjuncts to improve oxygenation:** | | | |
| Prone positioning | 9 | 0.13 | Appropriate |
| Recruitment manoeuvres | 8 | 0.09 | Appropriate |
| Inhaled prostacyclin analogues | 6.5 | 0.56 | Appropriate |
| Inhaled nitric oxide | 7.5 | 0.28 | Appropriate |
| Neuromuscular blocking agents | 9 | 0.04 | Appropriate |
| Referral for venovenous extracorporeal membrane oxygenation, available on site | 8.5 | 0.13 | Appropriate |
| Referral for venovenous extracorporeal membrane oxygenation, requiring patient transfer to an external site | 8 | 0.21 | Appropriate |
| **For critically ill patients with burn inhalation injury and concomitant ARDS receiving mechanical ventilation and experiencing refractory hypoxaemia, please rate the appropriateness of the following adjuncts to improve oxygenation:** | | | |
| Prone positioning | 9 | 0.13 | Appropriate |
| Recruitment manoeuvres | 8 | 0.13 | Appropriate |
| Inhaled prostacyclin analogues | 7 | 0.22 | Appropriate |
| Inhaled nitric oxide | 7 | 0.22 | Appropriate |
| Neuromuscular blocking agents | 9 | 0.04 | Appropriate |
| Referral for venovenous extracorporeal membrane oxygenation, available on site | 8.5 | 0.13 | Appropriate |
| Referral for venovenous extracorporeal membrane oxygenation, requiring patient transfer to an external site away from the burns centre | 8 | 0.16 | Appropriate |
| **Chapter 6: Pharmacotherapy** | | | |
| **For patients with mild burn inhalation injury (e.g., graded 1 using the Abbreviated Injury Score or an equivalent grade on a similar scoring system) and without contraindication to any of the proposed agents below, please rate the appropriate of the following treatment adjuncts:** | | | |
| Nebulised heparin – 5,000 IU administered four hourly, to degrade fibrin casts | 5 | 1.52 | Uncertain |
| Nebulised heparin – 10,000 IU administered four hourly, to degrade fibrin casts | 3 | 0.65 | Inappropriate |
| Nebulised N-acetylcysteine, as a mucolytic | 5 | 1.70 | Uncertain |
| Nebulised sodium bicarbonate, as a mucolytic | 5 | 0.94 | Uncertain |
| Nebulised salbutamol (albuterol), for bronchodilation | 5 | 1.36 | Uncertain |
| Nebulised racemic epinephrine, for bronchodilation | 3 | 0.63 | Inappropriate |
| Prophylactic antibiotics | 2 | 0.13 | Inappropriate |
| Corticosteroids | 1 | 0.26 | Inappropriate |
| **For patients with moderate burn inhalation injury (e.g., graded 2 using the Abbreviated Injury Score or an equivalent grade on a similar scoring system) and without contraindication to any of the proposed agents below, please rate the appropriate of the following treatment adjuncts:** | | | |
| Nebulised heparin – 5,000 IU administered four hourly, to degrade fibrin casts | 7 | 0.59 | Appropriate |
| Nebulised heparin – 10,000 IU administered four hourly, to degrade fibrin casts | 5 | 0.95 | Uncertain |
| Nebulised N-acetylcysteine, as a mucolytic | 7 | 0.65 | Appropriate |
| Nebulised sodium bicarbonate, as a mucolytic | 5 | 0.95 | Uncertain |
| Nebulised salbutamol (albuterol), for bronchodilation | 7 | 0.65 | Appropriate |
| Nebulised racemic epinephrine, for bronchodilation | 5 | 0.32 | Uncertain |
| Prophylactic antibiotics | 2 | 0.13 | Inappropriate |
| Corticosteroids | 2 | 0.13 | Inappropriate |
| **For patients with severe burn inhalation injury (e.g., graded 3 or 4 using the Abbreviated Injury Score or an equivalent grade on a similar scoring system) and without contraindication to any of the proposed agents below, please rate the appropriate of the following treatment adjuncts:** | | | |
| Nebulised heparin – 5,000 IU administered four hourly, to degrade fibrin casts | 8 | 0.33 | Appropriate |
| Nebulised heparin – 10,000 IU administered four hourly, to degrade fibrin casts | 6 | 0.22 | Uncertain |
| Nebulised N-acetylcysteine, as a mucolytic | 6 | 0.35 | Uncertain |
| Nebulised sodium bicarbonate, as a mucolytic | 5 | 0.49 | Uncertain |
| Nebulised salbutamol (albuterol), for bronchodilation | 7 | 0.47 | Appropriate |
| Nebulised racemic epinephrine, for bronchodilation | 5 | 0.27 | Uncertain |
| Prophylactic antibiotics | 2 | 0.29 | Inappropriate |
| Corticosteroids | 2 | 0.29 | Inappropriate |
