# Supplementary figures and images for: An international RAND/UCLA expert panel to determine the optimal diagnosis and management of burn inhalation injury
Source: Crit Care. 2023 Nov 27;27:459. doi: 10.1186/s13054-023-04718-w (PMC10680253; doi:10.1186/s13054-023-04718-w)

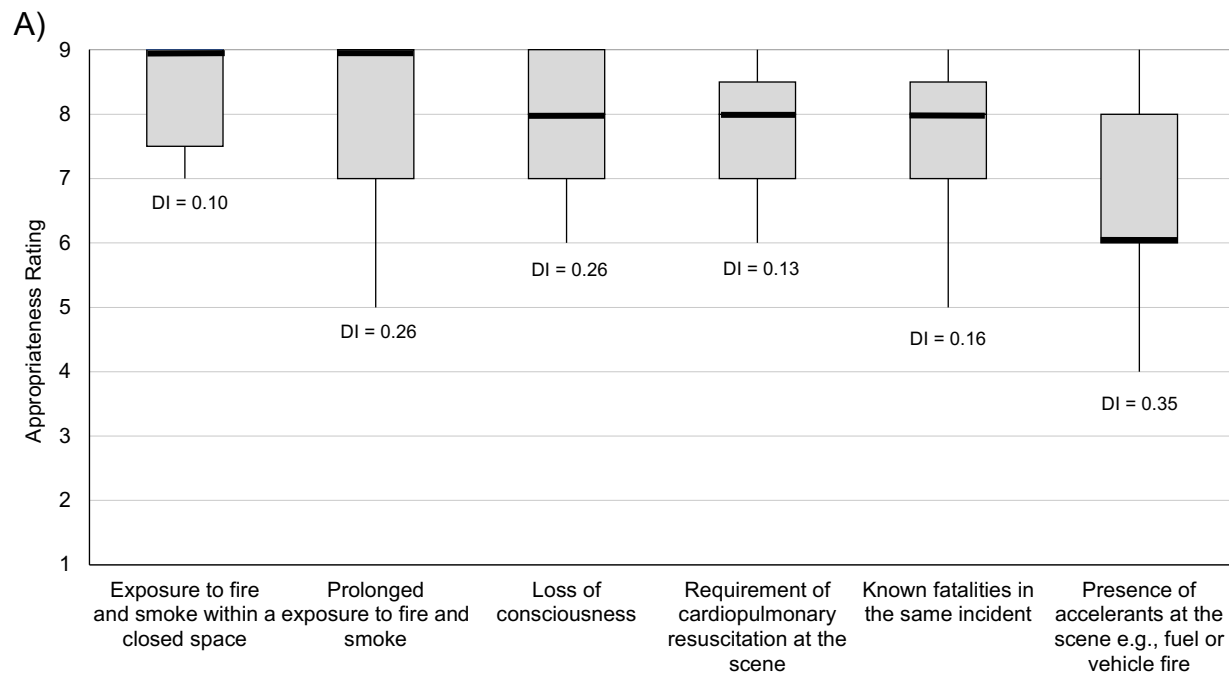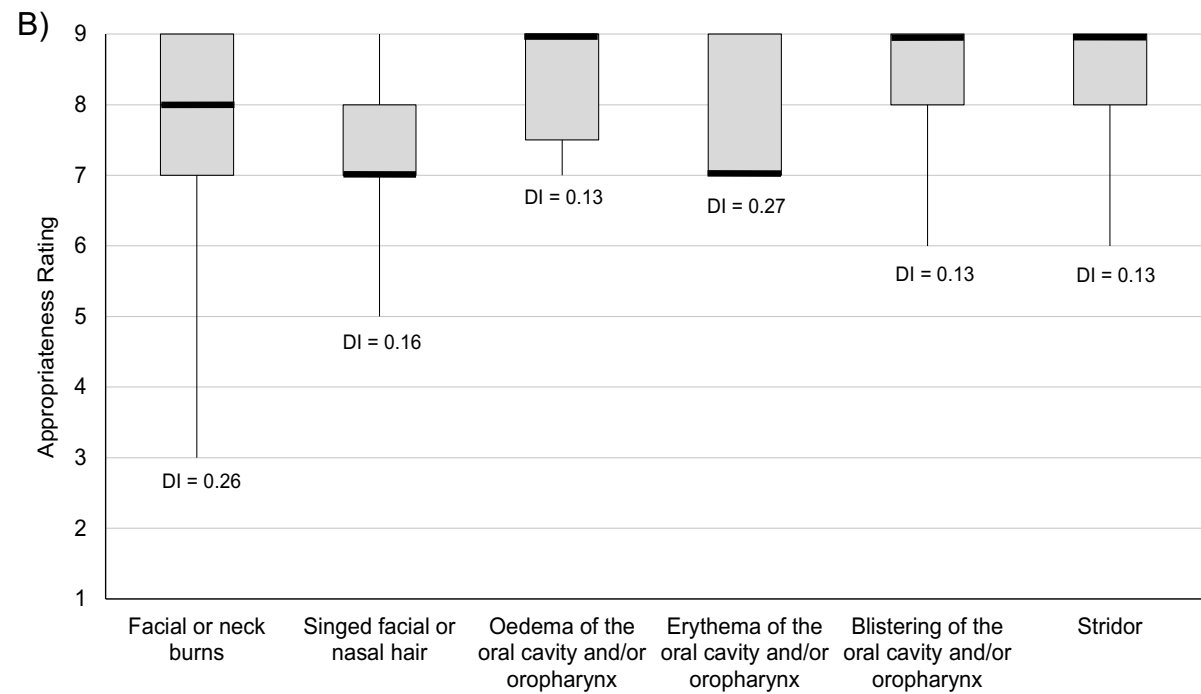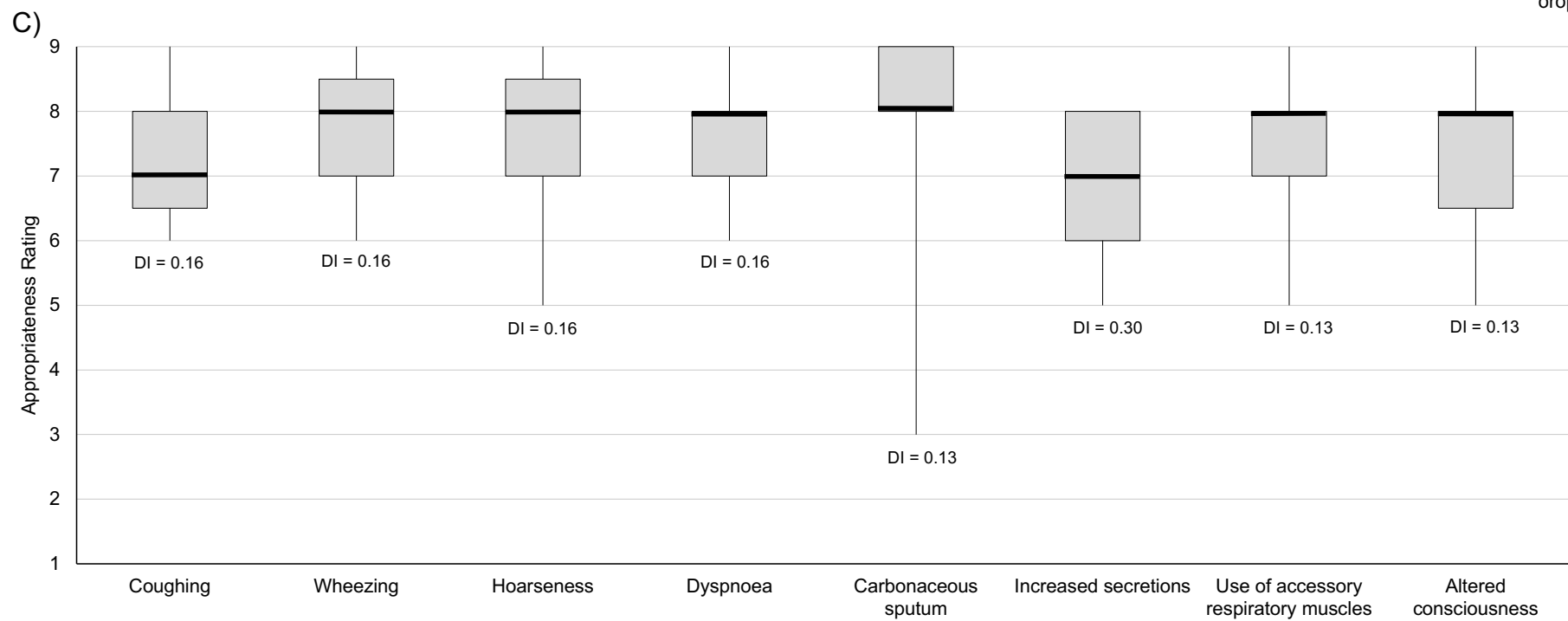

Supplement: Supplementary file 5 — Additional file 5: Fig. S2. Appropriateness of using history and examination findings as indicators of potential burn inhalation injury. A Indicators of potential burn inhalation injury. B Indicators of potential supraglottic thermal injury. C Indicators of potential subglottic and alveolar chemical injury. Median ratings are presented (bold black line in each box) with the interquartile range (edges of box) and maximum and minimum ratings (extending vertical lines). Statements with median ratings of 1–3 were classed as inappropriate, 4–6 as uncertain and 7–9 as appropriate. Disagreement (disagreement index ≥ 1) was not present for any statements. Panellists n = 15. DI, disagreement index. [file 13054_2023_4718_MOESM5_ESM.pdf]

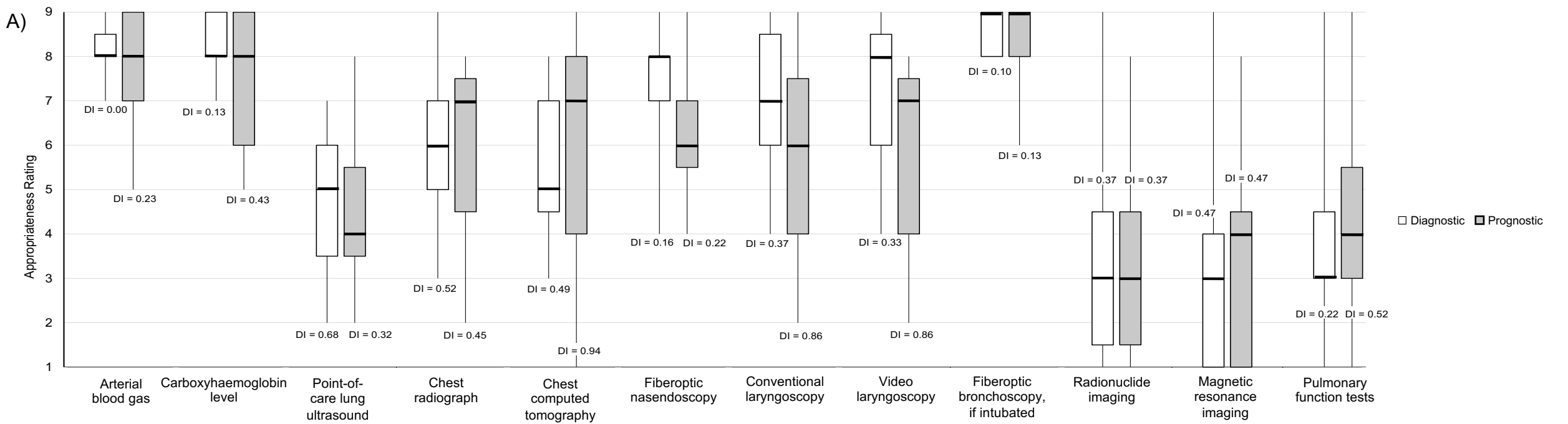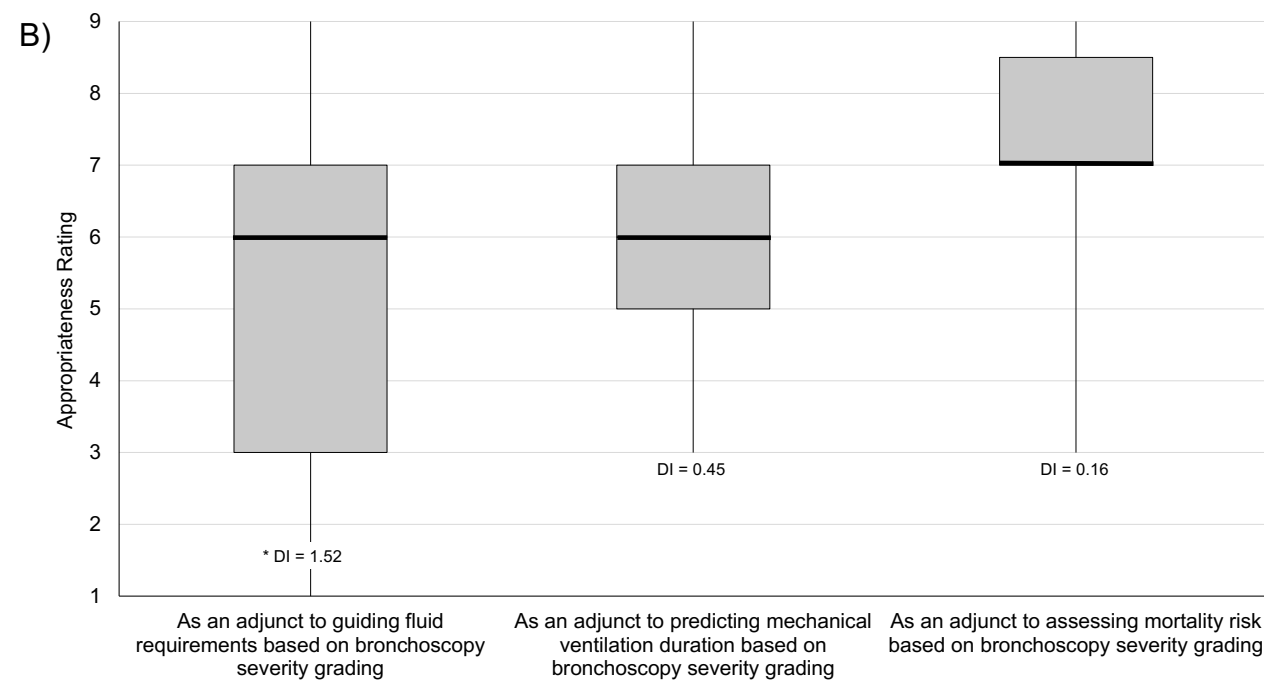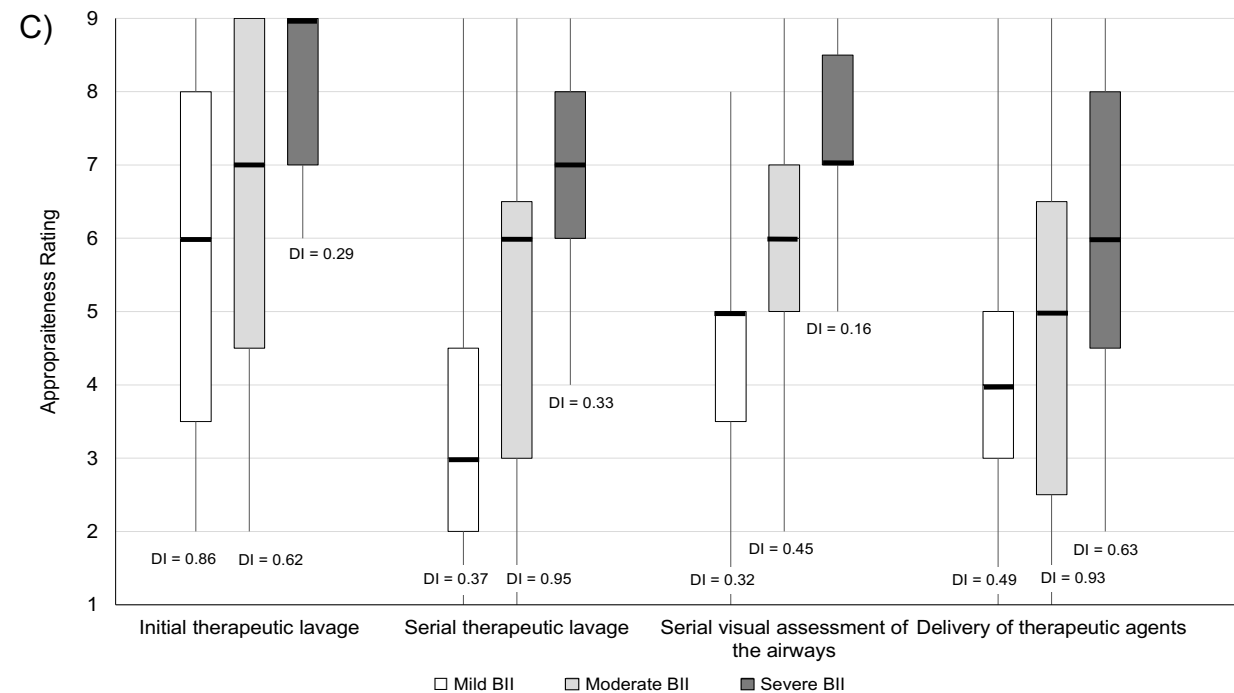

Supplement: Supplementary file 6 — Additional file 6: Fig. S3. Appropriateness of investigations in the management of burn inhalation injury. A Investigations as diagnostic and prognostic tools for burn inhalation injury. B Using diagnostic fiberoptic bronchoscopy to predict outcomes in burn inhalation injury. C Using therapeutic fiberoptic bronchoscopy for varying severities of burn inhalation injury. Median ratings are presented (bold black line in each box) with the interquartile range (edges of box) and maximum and minimum ratings (extending vertical lines). Statements with median ratings of 1–3 were classed as inappropriate, 4–6 as uncertain and 7–9 as appropriate. *Disagreement (disagreement index ≥ 1) was present for one statement, which was classed as uncertain. Burn inhalation injury severity was defined according to Abbreviated Injury Score criteria as mild (grade 1), moderate (grade 2) and severe (grades 3–4). Panellists n = 15. BII, burn inhalation injury; DI, disagreement index. [file 13054_2023_4718_MOESM6_ESM.pdf]

A)

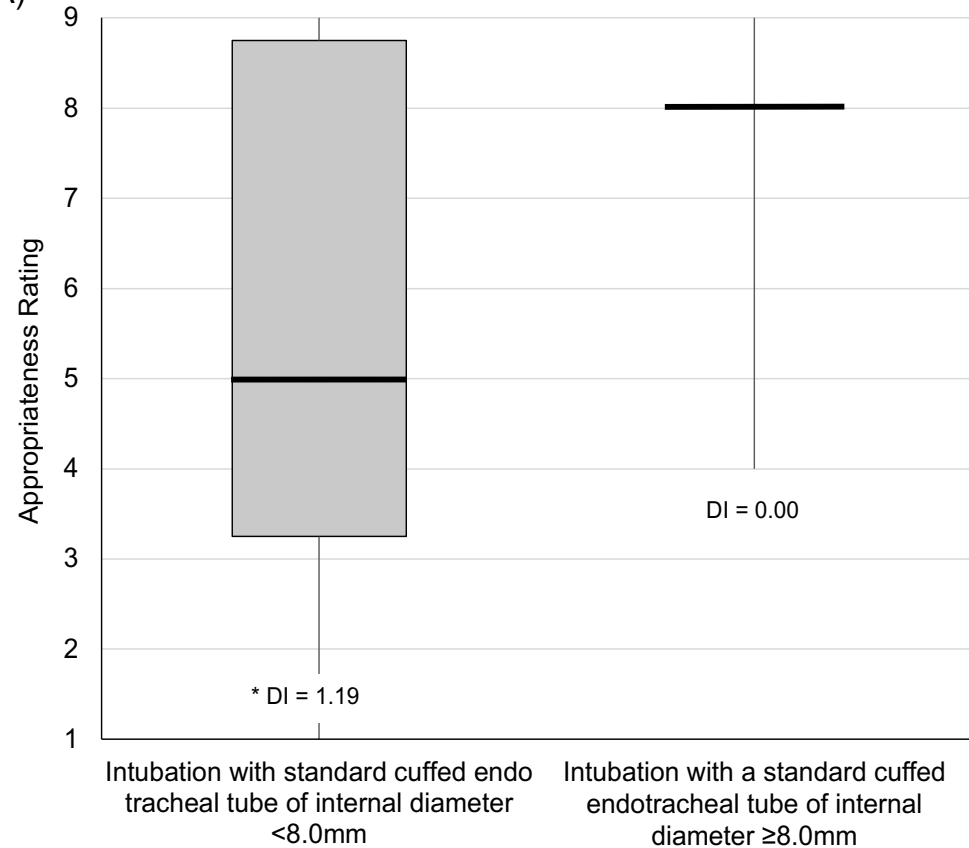

B)

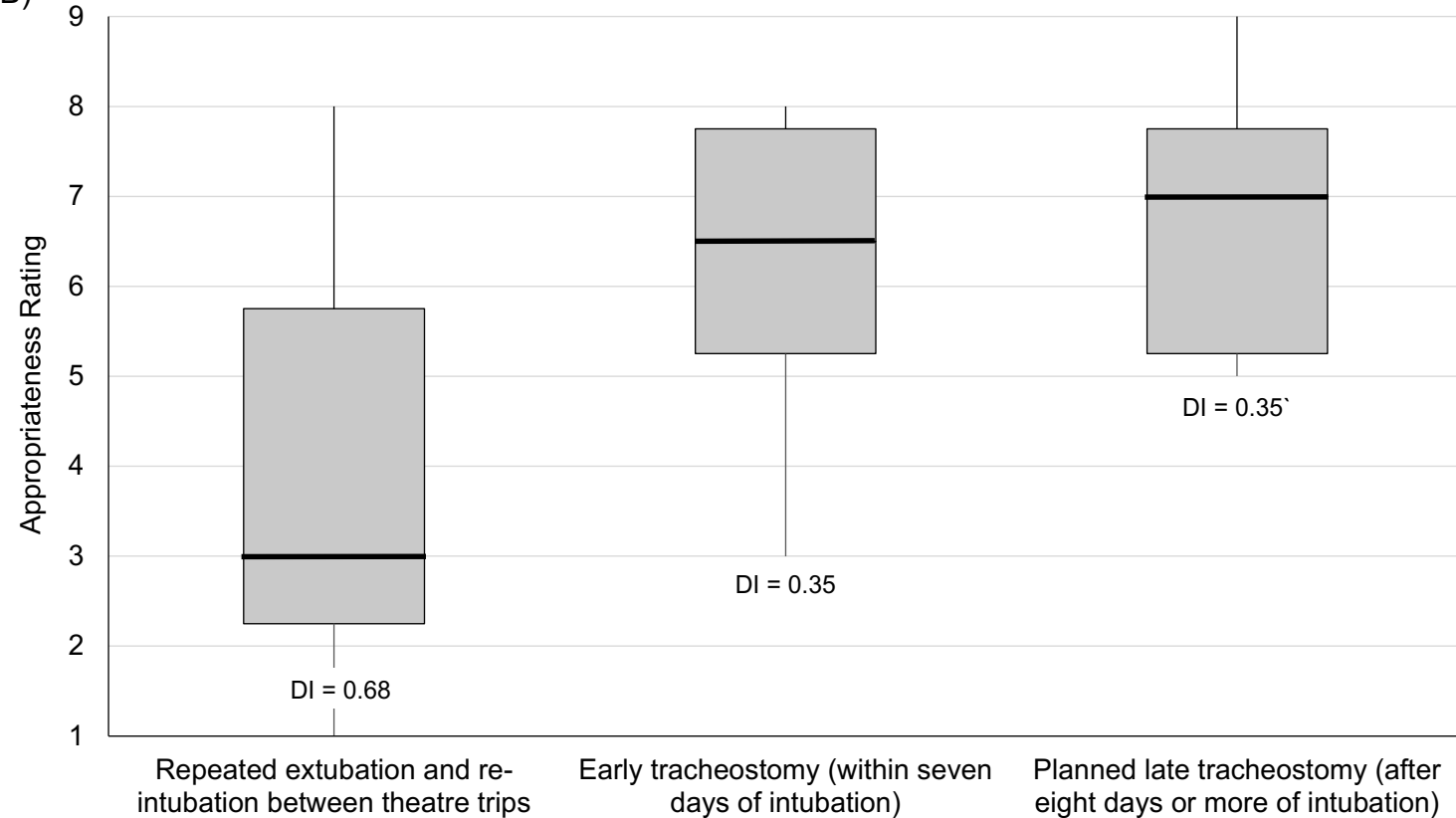

Supplement: Supplementary file 7 — Additional file 7: Fig. S4. Appropriateness of airway management strategies for burn inhalation injury. A Airway management strategies for burn inhalation injury patients at risk of airway compromise. B Airway management strategies for burn inhalation injury patients anticipated to require endotracheal intubation exceeding seven days. Median ratings are presented (bold black line in each box) with the interquartile range (edges of box) and maximum and minimum ratings (extending vertical lines). Statements with median ratings of < 3.5 were classed as inappropriate, ≥ 3.5 and < 6.5 as uncertain and ≥ 6.5 as appropriate. *Disagreement (disagreement index ≥ 1) was present for one statement, which was classed as uncertain. Panellists n = 10. DI, disagreement index. [file 13054_2023_4718_MOESM7_ESM.pdf]

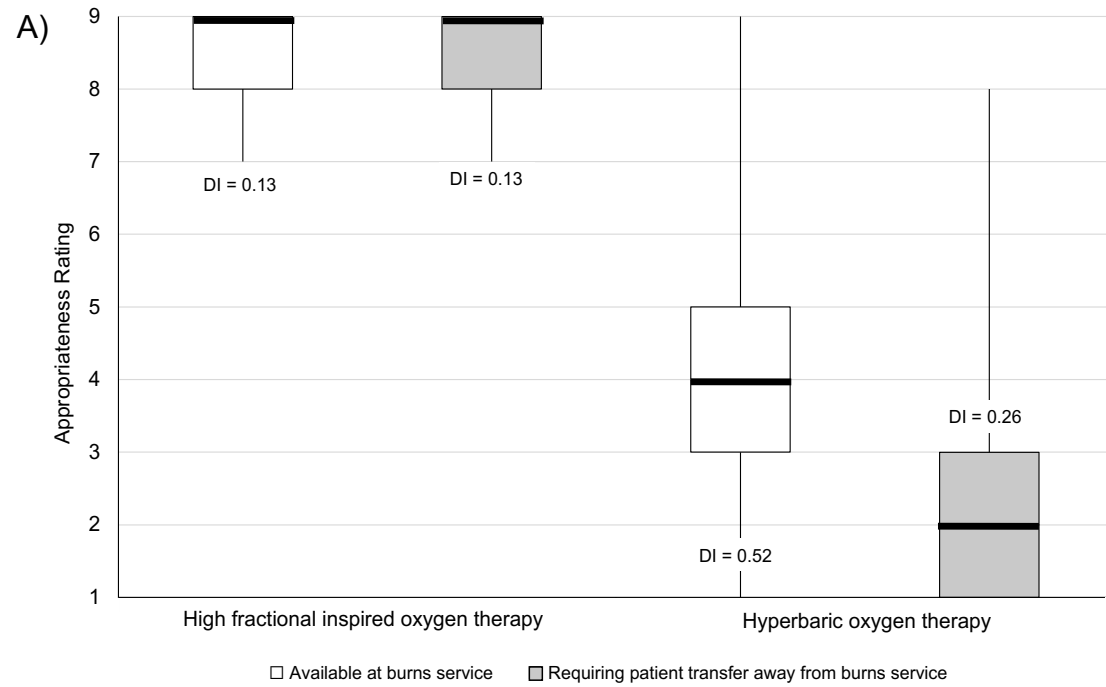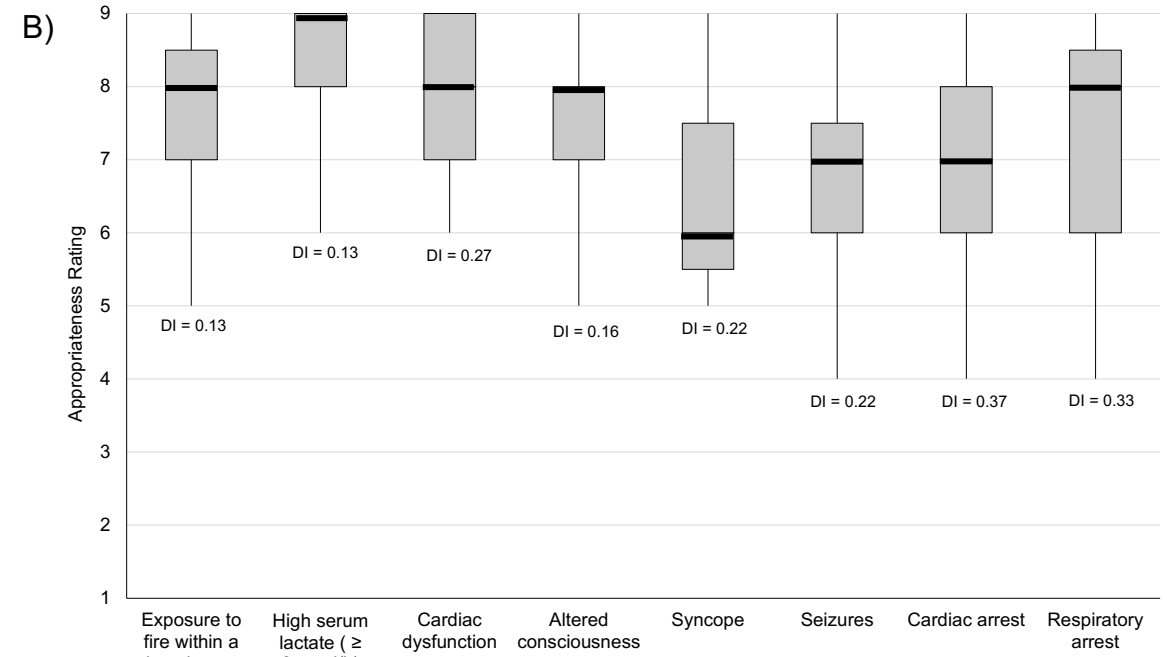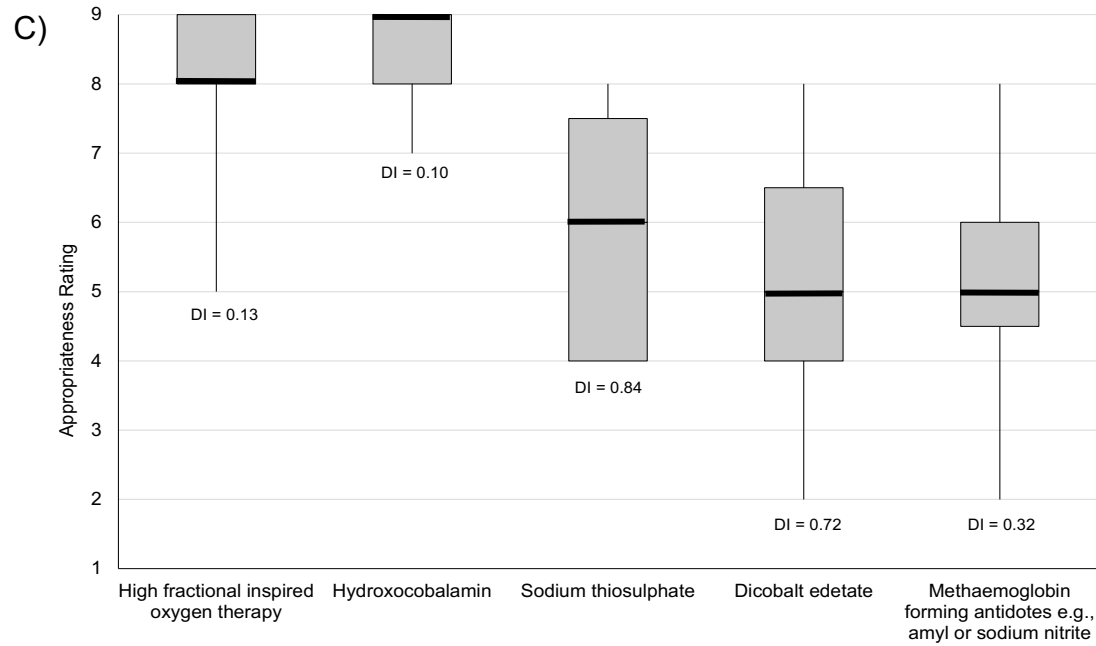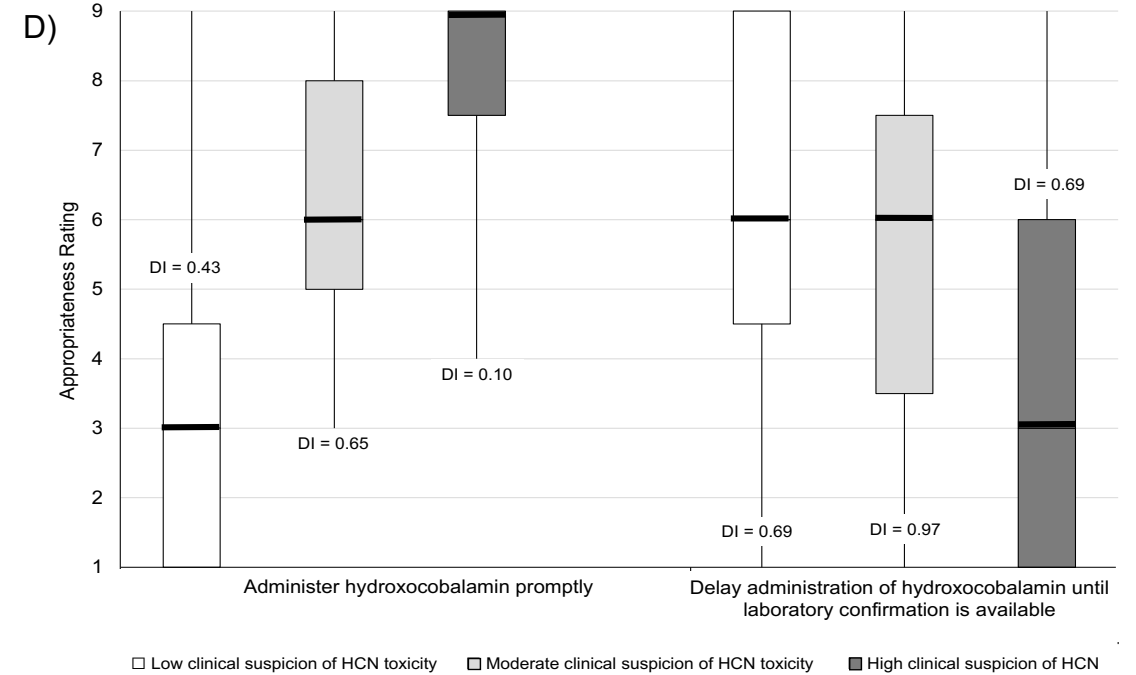

Supplement: Supplementary file 8 — Additional file 8: Fig. S5. Appropriateness of diagnostic and management strategies for burn inhalation injury associated systemic toxicity. A Management strategies for burn inhalation injury associated carbon monoxide toxicity. B Indicators of burn inhalation injury associated hydrogen cyanide toxicity. C Management strategies for burn inhalation injury associated hydrogen cyanide toxicity. D Timing of hydroxocobalamin administration for varying severities of burn inhalation injury. Median ratings are presented (bold black line in each box) with the interquartile range (edges of box) and maximum and minimum ratings (extending vertical lines). Statements with median ratings of 1–3 were classed as inappropriate, 4–6 as uncertain and 7–9 as appropriate. Disagreement (disagreement index ≥ 1) was not present for any statements. Clinical suspicion of hydrogen cyanide toxicity was defined as low (normal blood lactate and the absence of potentially suspicious features), moderate (moderate lactatemia below 8 mmol/L and few potentially suspicious features) and high (hyperlactataemia ≥ 8 mmol/L and potentially suspicious features including anion gap lactic metabolic acidosis, altered consciousness, unexplained cardiac dysfunction). Panellists n = 15. DI, disagreement index; HCN, hydrogen cyanide. [file 13054_2023_4718_MOESM8_ESM.pdf]

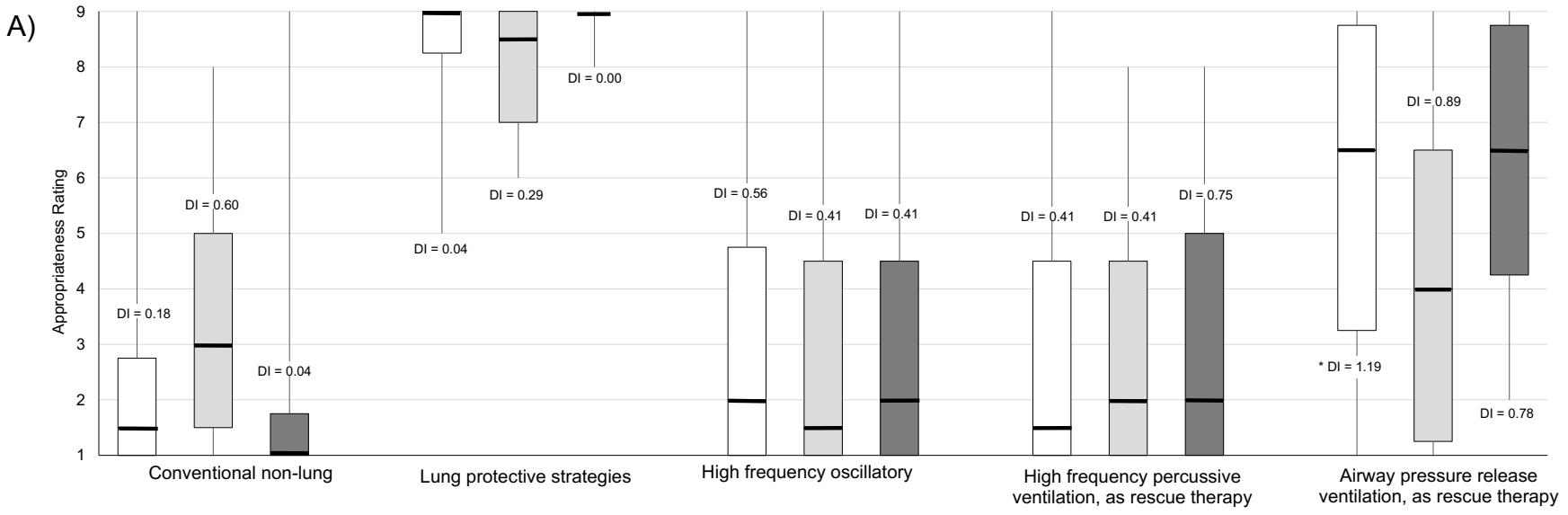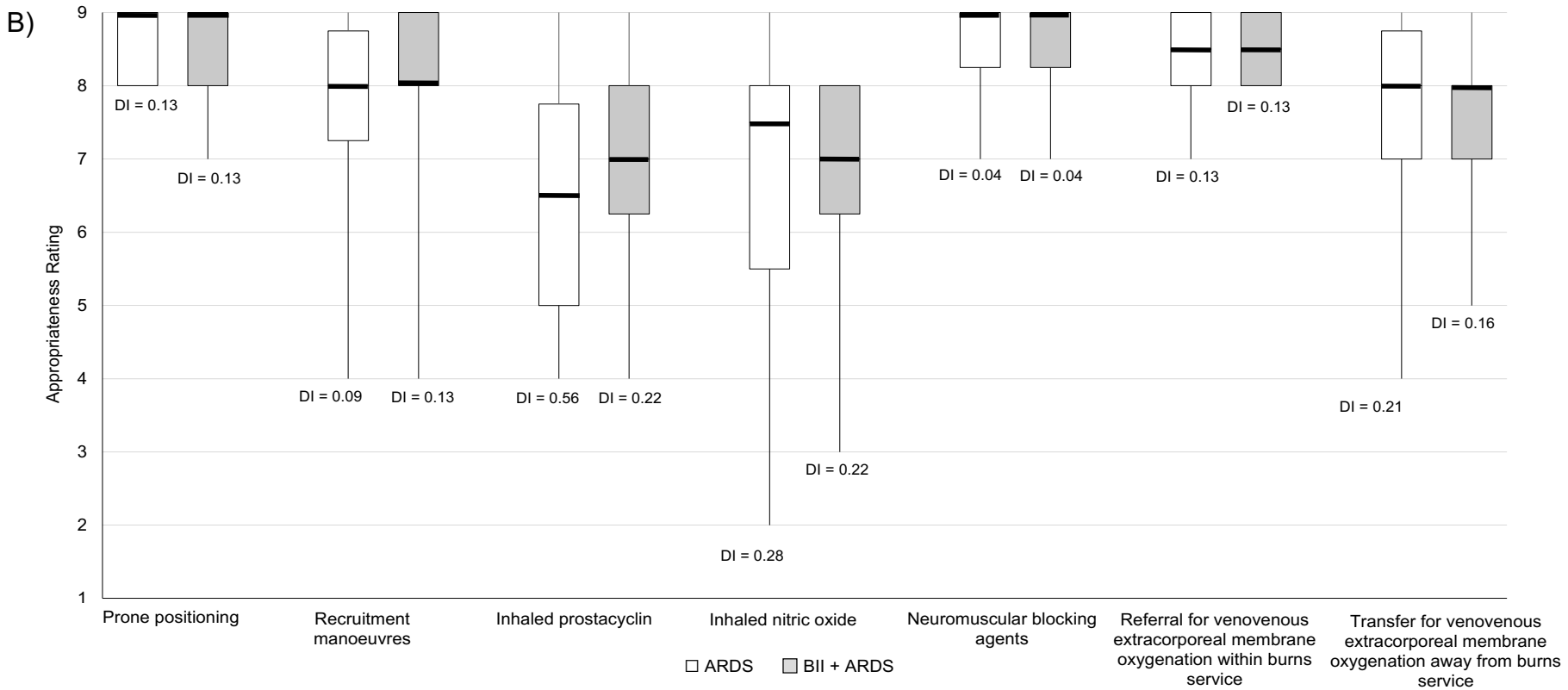

Supplement: Supplementary file 9 — Additional file 9: Fig. S6. Appropriateness of ventilation strategies for burn inhalation injury and/or acute respiratory distress syndrome. A Invasive mechanical ventilation modalities for acute respiratory distress syndrome, burn inhalation injury or burn inhalation injury with concomitant acute respiratory distress syndrome. B Mechanical ventilation adjuncts for burn inhalation injury patients with refractory hypoxaemia. Median ratings are presented (bold black line in each box) with the interquartile range (edges of box) and maximum and minimum ratings (extending vertical lines). Statements with median ratings of < 3.5 were classed as inappropriate, ≥ 3.5 and < 6.5 as uncertain and ≥ 6.5 as appropriate. *Disagreement (disagreement index ≥ 1) was present for one statement, which was classed as uncertain. Lung protective ventilatory strategies = tidal volume < 6 mL/kg ideal body weight, plateau pressure < 30 cmH20. Panellists n = 10. ARDS, acute respiratory distress syndrome; BII, burn inhalation injury; DI, disagreement index. [file 13054_2023_4718_MOESM9_ESM.pdf]

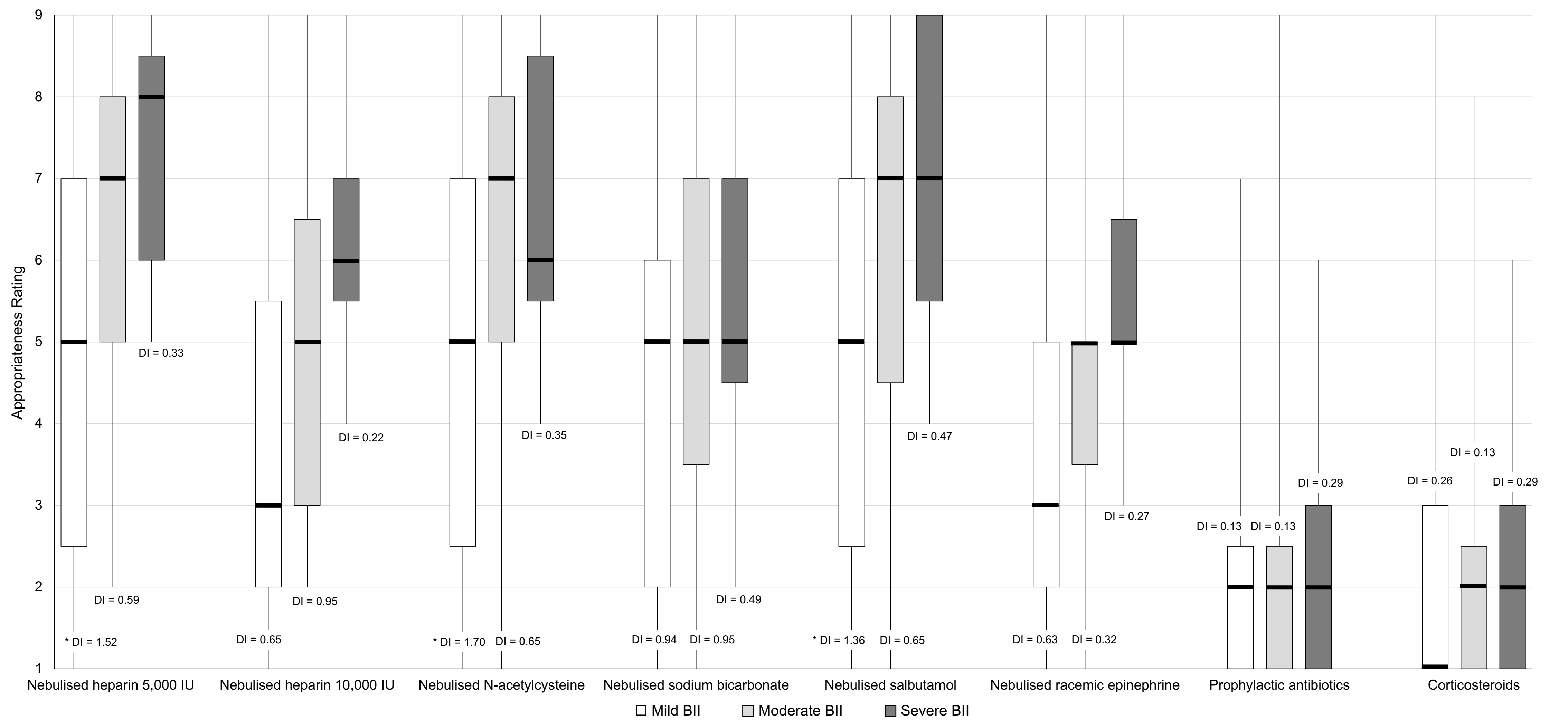

Supplement: Supplementary file 10 — Additional file 10: Fig. S7. Appropriateness of pharmacological therapies for varying severities of burn inhalation injury. Median ratings are presented (bold black line in each box) with the interquartile range (edges of box) and maximum and minimum ratings (extending vertical lines). Statements with median ratings of 1–3 were classed as inappropriate, 4–6 as uncertain and 7–9 as appropriate. *Disagreement (disagreement index ≥ 1) was present for three statements, which were classed as uncertain. Burn inhalation injury severity was defined according to Abbreviated Injury Score criteria as mild (grade 1), moderate (grade 2) and severe (grades 3–4). Panellists n = 15. BII, burn inhalation injury; DI, disagreement index; IU, international units. [file 13054_2023_4718_MOESM10_ESM.pdf]
